# Estrogen receptor-α ablation reverses muscle fibrosis and inguinal hernias

**DOI:** 10.1172/JCI179137

**Published:** 2025-02-04

**Authors:** Tanvi Potluri, Tianming You, Ping Yin, John Coon, Jonah J. Stulberg, Yang Dai, David J. Escobar, Richard L. Lieber, Hong Zhao, Serdar E. Bulun

**Affiliations:** 1 Department of Obstetrics & Gynecology, Feinberg School of Medicine, Northwestern University, Chicago, Illinois, USA.; 2 Department of Surgery, McGovern Medical School at the University of Texas Health Sciences Center, Houston, Texas, USA.; 3 Department of Biomedical Engineering, University of Illinois Chicago, Chicago, Illinois, USA.; 4 Department of Pathology, Feinberg School of Medicine, and; 5 Departments of Physical Medicine and Rehabilitation and Biomedical Engineering, Northwestern University, Chicago, Illinois, USA.; 6 Research Service, Hines VA Medical Center, Maywood, Illinois, USA.; 7 Shirley Ryan AbilityLab, Chicago, Illinois, USA.

**Keywords:** Cell biology, Muscle biology, Reproductive biology, Fibrosis, Sex hormones, Skeletal muscle

## Abstract

Fibrosis of the lower abdominal muscle (LAM) contributes to muscle weakening and inguinal hernia formation, an ailment that affects a noteworthy 50% of men by age 75 and necessitates surgical correction as the singular therapy. Despite its prevalence, the mechanisms driving LAM fibrosis and hernia development remain poorly understood. Using a humanized mouse model that replicates the elevated skeletal muscle tissue estrogen concentrations seen in aging men, we identified estrogen receptor-α (ESR1) as a key driver of LAM fibroblast proliferation, extracellular matrix deposition, and hernia formation. Fibroblast-specific ESR1 ablation effectively prevented muscle fibrosis and herniation, while pharmacological ESR1 inhibition with fulvestrant reversed hernias and restored normal muscle architecture. Multiomics analyses of in vitro LAM fibroblasts from humanized mice unveiled an estrogen/ESR1-mediated activation of a distinct profibrotic cistrome and gene expression signature, concordant with observations in inguinal hernia tissues in human males. Our findings hold significant promise for prospective medical interventions targeting fibrotic conditions and present non-surgical avenues for addressing inguinal hernias.

## Abstract 2

Activation of estrogen receptor-alpha (ESR1) in mouse fibroblasts within lower abdominal muscles stimulates fibrosis, leading to inguinal hernias, which can be fully reversed by inhibiting the ESR1 pathway.

## Introduction

Inguinal hernias, characterized by the protrusion of intestinal viscera through weakened lower abdominal muscles (LAMs) in the groin area, represent a significant health concern, with 1 in 2 men anticipated to develop the condition by the age of 75 and 10%–15% experiencing recurrent hernias (1). Current treatment relies on surgical repair, which introduces additional complexities, especially in older individuals and resource-poor settings (1–4). Consequently, inguinal hernias pose a substantial global public health challenge. Despite their prevalence, the mechanisms that instigate hernia formation remain poorly understood, necessitating fundamental research in hernia biology.

The humanized aromatase mouse model (*Arom^hum^*) offers a unique opportunity to unravel the molecular mechanisms of herniation by enhancing local testosterone-to-estradiol (E2) conversion via the aromatase enzyme in skeletal muscles, similar to the process in aging humans (5, 6). *Arom^hum^* mice, expressing the human aromatase gene (*CYP19A1*), mimic human aromatase activity across various mouse tissues, facilitating localized E2 synthesis in the LAM (6, 7). This E2 production triggers LAM fibroblast activation, resulting in fibrotic skeletal muscles, a hallmark of scrotal hernias in these mice (6). E2 typically binds to 3 estrogen receptors (ESRs) — ESR-α (ESR1), ESR2, and G protein–coupled estrogen receptor 1 (GPER1) — to exert its biological functions (8). Our prior investigation revealed a higher expression of ESR1, as opposed to ESR2 or GPER1, in LAM fibroblasts in *Arom^hum^* mice compared with wild-type (WT) mice (6, 9). Additionally, this high ESR1 expression in the fibroblasts is unique to the LAMs compared with other muscle groups, such as the upper abdominal muscles and the quadriceps (6, 9). However, the necessity and role of E2/ESR1 signaling in the LAM and inguinal hernia formation remained unclear.

Single-cell RNA sequencing (scRNA-Seq) analysis of LAM further delineated a hernia-associated fibroblast (HAF) cluster in *Arom^hum^* mice, marked by elevated *ESR1* expression. HAFs exhibit characteristics of highly activated pathological fibroblasts, demonstrating increased fibroblast proliferation capacity and extracellular matrix (ECM) remodeling. Additionally, HAFs highly express the fibro-adipogenic progenitor (FAP) marker platelet-derived growth factor-α (*PDGFRA*) (9). FAPs are muscle-resident multipotent mesenchymal stem cells that can differentiate into adipocytes, fibroblasts, or osteocytes (10, 11). While FAPs usually contribute to tissue regeneration during repair, dysregulation in conditions such as dystrophies or chronic injuries leads to excessive ECM deposition and fibrosis (12–15). FAPs or PDGFRA^+^ fibroblasts have been implicated in various skeletal muscle pathologies, contributing to fibrosis development (11–19). Thus, we hypothesize that HAFs, a subset of PDGFRA^+^ fibroblasts (FAPs), possibly activated by E2/ESR1 signaling, play an essential role in hernia formation in *Arom^hum^* mice (9).

In this study, we demonstrate the prevention and reversal of LAM fibrosis and herniation through genetic ablation and pharmacological inhibition of E2/ESR1-signaling HAFs. We further characterize LAM fibrosis through multiomics analyses of E2/ESR1 action on the HAFs to better understand molecular mechanisms underlying hernia formation. Our findings have significant implications for developing novel pharmacological treatments for inguinal hernias and therapies to prevent or reverse fibrosis in skeletal muscle and other tissues.

## Results

### Fibroblast-specific ablation of ESR1 prevents herniation in Arom^hum^ mice.

**Figure 1:**
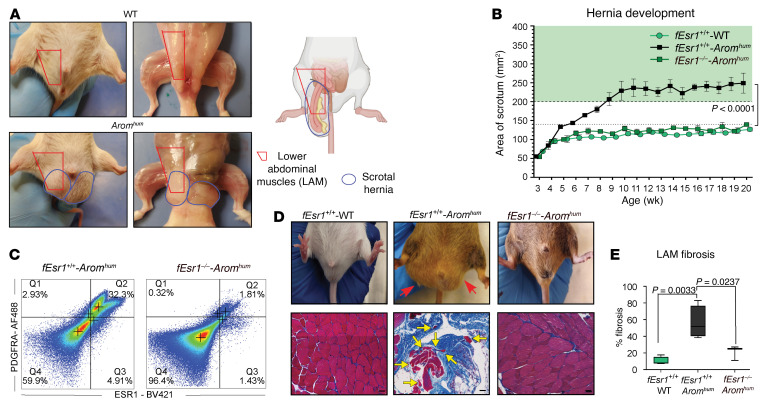
Fibroblast-specific ablation of ESR1 in *Arom^hum^* mice prevents herniation. (**A**) Representative images of WT and *Arom^hum^* mice, and an illustration depicting scrotal hernia and LAMs. Created with BioRender (biorender.com). (**B**) Measurement of scrotal hernia size with age in fibroblast-specific *Esr1*-knockout mice (*fEsr1^–/–^* *Arom^hum^*) and *fEsr1^+/+^* *Arom^hum^* and *fEsr1^+/+^* WT littermate controls (*n* = 3–4 per group, mean ± SEM, repeated-measures ANOVA with Bonferroni multiple comparisons). (**C**) Flow cytometry dot plots showing the percentage of PDGFRA^+^ estrogen receptor-α–positive HAFs in LAMs from *fEsr1^–/–^* *Arom^hum^* and control *fEsr1^+/+^* *Arom^hum^* mice (*n* = 3). (**D**) Representative images of scrotal hernias (top) and Masson’s trichrome–stained LAMs (bottom). Red arrows point to scrotal hernia, while yellow arrows point to atrophied myofibers. Scale bars: 100 μm. (**E**) Quantification of the fibrotic area in *fEsr1^–/–^* *Arom^hum^*, *fEsr1^+/+^* *Arom^hum^*, and *fEsr1^+/+^* WT mice (*n* = 3–4 per group, median ± interquartile range, 1-way ANOVA with Bonferroni multiple comparisons).

All *Arom^hum^* mice developed a fibrotic process characterized by the proliferation of fibroblasts depositing excess ECM in the LAM, weakened muscle tissue, and formation of scrotal hernias — with hernia sacs containing abdominal viscera, gonads, gonadal fat, and urinary bladder (Figure 1A). Primary fibroblasts isolated from *Arom^hum^* LAM confirmed the coexpression of both ESR1 and PDGFRA proteins, validating their identity as HAFs (9). This expression pattern aligns with our previous single-cell RNA study of *Arom^hum^* LAM (Supplemental Figure 1, A and B; supplemental material available online with this article; https://doi.org/10.1172/JCI179137DS1) (9). To discern the impact of ESR1 signaling on LAM HAFs and hernia development, we engineered a fibroblast-specific ESR1 knockout in *Arom^hum^* mice (*fEsr1^–/–^ Arom^hum^*) by cross-breeding floxed ESR1, PDGFRA-cre, and *Arom^hum^* mice. Notably, the *fEsr1^–/–^ Arom^hum^* mice did not show hernia formation during a 20-week observation period (Figure 1B). Similarly, the WT control mice (*fEsr1^+/+^* WT) displayed no herniation (Figure 1B). As expected, all *fEsr1^+/+^ Arom^hum^* (i.e., *Arom^hum^*) littermate controls displayed hernia onset at approximately 5 weeks of age, with hernia sizes increasing over time (Figure 1, B and D). The percentage of HAFs marked by expression of PDGFRA and ESR1 was significantly lower in the hernia-free *fEsr1^–/–^ Arom^hum^* mice compared with the positive control herniated *fEsr1^+/+^ Arom^hum^* mice (1.8% vs. 32.3%; Figure 1C). This marked reduction in ESR1 expression in PDGFRA-cre–driven *Esr1^fl/fl^* mice serves as a crucial validation of our model, confirming the specificity of the ESR1 knockout in targeting HAFs. Furthermore, ESR1 depletion in LAM HAFs effectively prevented LAM fibrosis and muscle atrophy, as corroborated by Masson’s trichrome staining from *fEsr1^–/–^ Arom^hum^* mice (Figure 1, D and E). In contrast, *fEsr1^+/+^ Arom^hum^* mice exhibited significantly higher LAM fibrosis than both ESR1-depleted *fEsr1^–/–^ Arom^hum^* mice and the *fEsr1^+/+^* WT controls (Figure 1, D and E). These findings underscore the central role of ESR1 signaling in the stimulation and expansion of LAM HAFs to drive scrotal herniation in *Arom^hum^* mice.

### Inhibition of E2/ESR1 signaling prevents and reverses hernias in Arom^hum^ mice.

**Figure 2:**
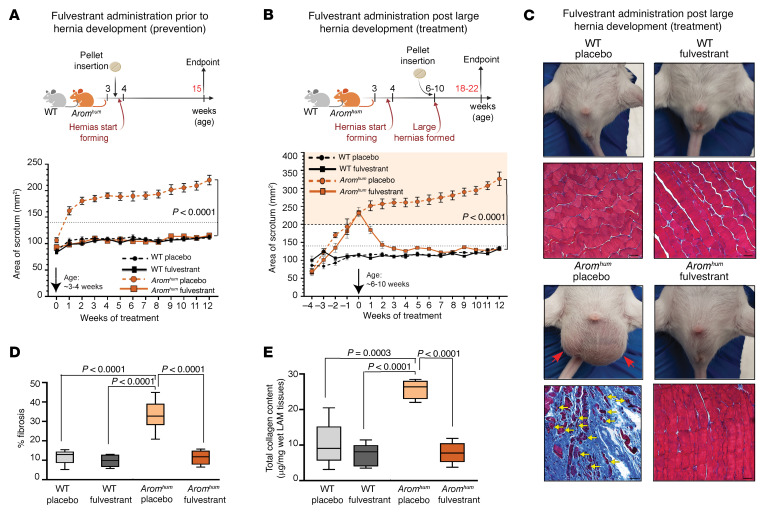
Fulvestrant treatment prevents and reverses well-established large hernias in mice. (**A**) Schematic of hernia prevention study design (top) and measurement of scrotal hernias (bottom); fulvestrant was administered before hernia formation. Arrow indicates the week of pellet implantation (*n* = 10–15 per group, mean ± SEM, repeated-measures ANOVA). (**B**) Schematic of hernia treatment study design (top) and measurement of scrotal hernias (bottom); fulvestrant was administered after large hernias were formed. Arrow indicates the week of pellet implantation (*n* = 10–15 per group, mean ± SEM, repeated-measures ANOVA with Bonferroni multiple comparisons). In both **A** and **B**, the dotted line at 140 mm^2^ represents normal scrotum size before hernia development, and the orange shaded region represents large scrotal hernia size (>200 mm^2^). Created with BioRender (biorender.com). (**C**) Representative images of LAM morphology and Masson’s trichrome staining of LAMs from mice in the treatment study (**B**). Red arrows point to bilateral scrotal hernias in placebo-treated mice, while yellow arrows point to atrophied myofibers. (**D** and **E**) Quantification of the fibrotic area (**D**) and collagen content by hydroxyproline assay (**E**) in the mouse LAM treatment study (**B** and **C**) (*n* = 5–6 per group, median ± interquartile range, 2-way ANOVA with Bonferroni multiple comparisons; scale bars: 100 μm).

To explore pharmacological interventions for hernias, we used fulvestrant, an E2/ESR antagonist that competitively blocks E2 binding to ESRs, leading to subsequent ESR degradation. Analogously to *fEsr1^–/–^ Arom^hum^* mice in Figure 1, *Arom^hum^* mice given slow-release fulvestrant pellets at 3–4 weeks of age (prior to hernia formation) did not develop hernias, whereas all *Arom^hum^* mice given placebo pellets exhibited progressive hernia growth over time (Figure 2A). To explore the potential of fulvestrant to reverse established hernias, *Arom^hum^* mice with large hernias (>200 mm^2^) at approximately 6–10 weeks of age were treated with fulvestrant slow-release pellets. Remarkably, within 2 weeks, fulvestrant-treated mice displayed a significant reduction in hernia size (Figure 2B). Subsequently, all fulvestrant-treated mice exhibited complete hernia regression with scrotal sizes comparable to those of WT mice after 4 weeks of treatment (Figure 2C). Histological examination after 12 weeks of treatment demonstrated extensive fibrotic degeneration in the LAM of placebo-treated *Arom^hum^* mice, whereas those receiving fulvestrant displayed normal muscle tissue and no fibrosis (Figure 2, C and D, and Supplemental Figure 3A). Remarkably, within just 1 week, fulvestrant-treated mice exhibited a stalling of herniation (Supplemental Figure 2A). Although hernia regression was not achieved during this short period, subsequent histological and immunohistochemical analyses show reduction in fibrosis and increase in muscle regeneration (Supplemental Figure 3, B–D) (20, 21). Furthermore, collagen content in the LAM tissue of *Arom^hum^* mice after the longer 90-day fulvestrant treatment was comparable to the levels in WT mice (Figure 2E). These findings underscore the translational potential of fulvestrant as a pharmacological approach for both preventing and reversing hernias.

Subsequently, we administered raloxifene HCl — a partial antagonist of E2/ESR — to *Arom^hum^* mice harboring large scrotal hernias (>200 mm^2^). Raloxifene administration effectively reduced hernia sizes, with results similar to those obtained with fulvestrant (Figure 2B and Supplemental Figure 2B). Hernia size in placebo-treated *Arom^hum^* mice continued to increase to about 300 mm^2^ during treatment. However, a 10-week raloxifene treatment reduced hernia from large to small/medium sizes, suggesting stoichiometric effects of the partial E2/ESR antagonist raloxifene on hernia regression compared with the E2/ESR antagonist fulvestrant (Figure 2B and Supplemental Figure 2B). To ascertain the specificity of hernia regression to ESR1, we used methyl-piperidino-pyrazole (MPP), an ESR1-selective antagonist, in *Arom^hum^* mice with large scrotal hernias. A significant reduction in hernia sizes was evident following a 21-day treatment regimen (Supplemental Figure 2C). Despite a significant reduction in hernia sizes, the effects of MPP were relatively partial compared with those of fulvestrant treatment. This disparity may be attributed to MPP’s short half-life and its pharmacokinetic properties, which might have limited its therapeutic efficacy. In contrast, ESR1 deletion and fulvestrant treatment provide more comprehensive and stronger inhibition of ESR1 action, leading to more pronounced effects on hernia regression. The ESR2- and GPER1-selective antagonists PHTPP and G-15, respectively, exhibited no discernible effects on hernia size compared with placebo treatment (Supplemental Figure 2, D and E). These findings underscore the predominant role of ESR1 as the primary ESR driving and regressing herniation in *Arom^hum^* mice.

### E2 or fulvestrant modifies chromatin accessibility, its occupancy by ESR1, and the transcriptome in HAFs.

**Figure 3:**
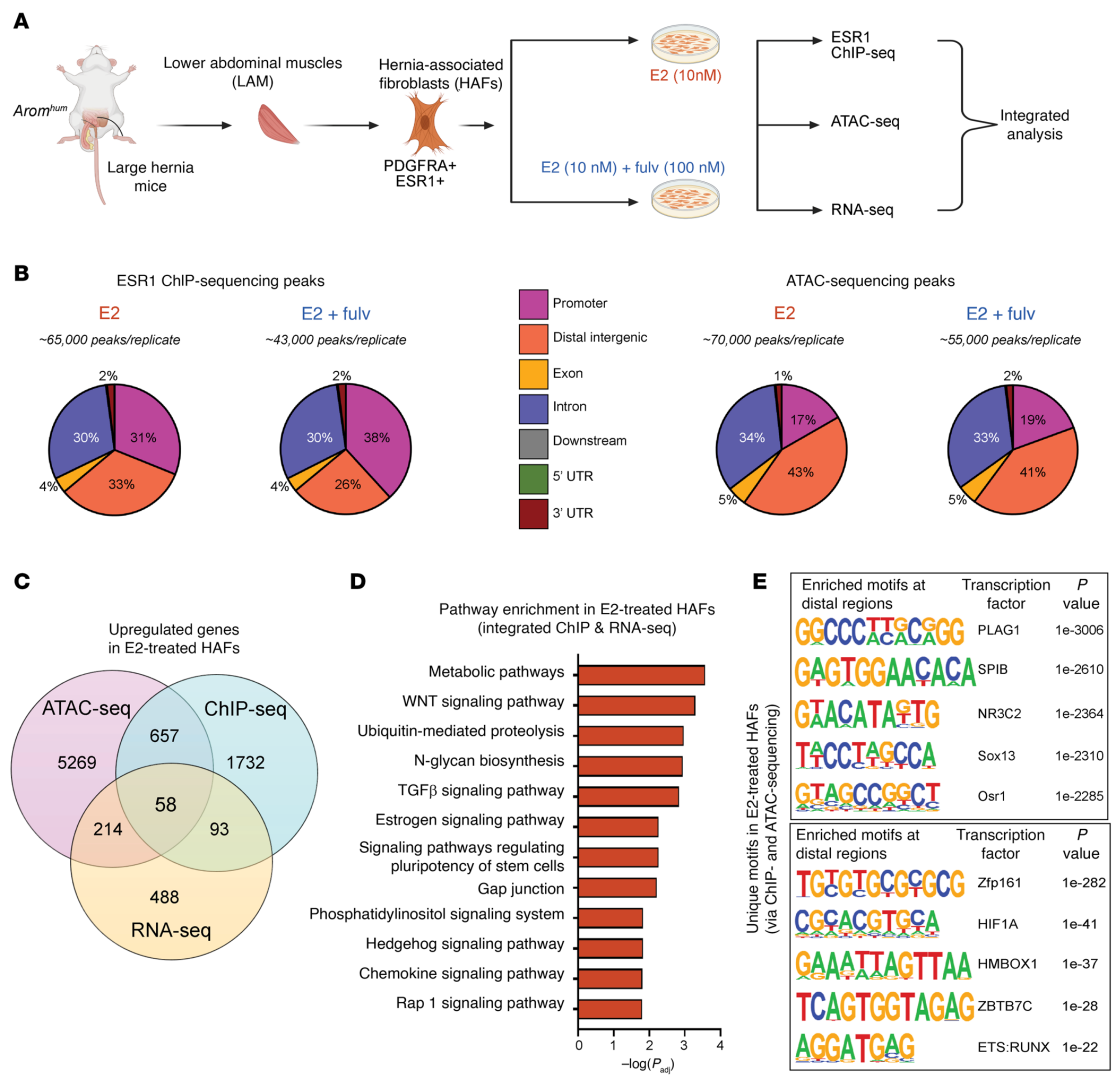
Multiomics analysis reveals E2/ESR1 signaling changes in HAFs. (**A**) Illustration of experimental design for multiomics studies. Created with BioRender (biorender.com). (**B**) Genomic distribution of ESR1 binding events in ChIP-Seq and open chromatin peaks in ATAC-seq in HAFs after E2 or E2 plus fulvestrant treatment (*n* = 3 per group). (**C**) Venn diagram showing overlap of genes upregulated with E2 treatment compared with E2 plus fulvestrant treatment in multiomics assays: RNA-Seq, ChIP-Seq, and ATAC-seq (fold change > 1.2, *P* < 0.05). (**D**) Significantly upregulated pathways in HAFs after E2 treatment. (**E**) Unique motifs enriched at the promoter and distal regions from both ChIP-Seq and ATAC-seq after E2 treatment.

We investigated the genome-wide, epigenomic, and transcriptomic effects of E2 and fulvestrant in HAFs to reveal underlying mechanisms responsible for LAM fibrosis and herniation. Based on our earlier scRNA-Seq and flow cytometry results, HAFs make up 50%–80% of the fibroblasts present in LAM (9). To ensure a higher purity of HAF populations, we used a preplating procedure, allowing us to selectively obtain adherent, pathogenic fibroblasts while minimizing the presence of other cell types. By the second passage, our cultures consistently contained only HAFs, as confirmed by immunostaining for ESR1 and PDGFRA (Figure 1B). This preplating method effectively excluded myogenic cells and other nonfibroblast populations, ensuring the purity of our HAF cultures. Notably, we observed that HAFs cultured in serum-rich or E2-supplemented conditions exhibited rapid proliferation, higher viability, and increased secretion of ECM, further underscoring their pathogenic role in fibrosis. HAFs treated with E2 alone or E2 with fulvestrant were subjected to multiomics analyses (Figure 3A). ChIP-Seq using an antibody against ESR1 revealed higher ESR1 binding in distal intergenic regions, suggesting a critical influence of enhancer regions on transcriptional regulation (Figure 3B, Supplemental Figure 5, A and B, and Supplemental Figure 6A). In contrast, the E2/ESR antagonist fulvestrant decreased total ESR1 binding and accessible chromatin regions (Figure 3B, Supplemental Figure 5, A and B, and Supplemental Figure 6A). RNA-Seq revealed distinct transcriptomic changes induced by E2 treatment, including the upregulation of pathways related to mesenchymal cell proliferation, ECM organization, and TGF-β/WNT signaling pathways (Supplemental Figure 7, A, B, and D). In contrast, fulvestrant binding to ESR1 downregulated these E2-driven effects with marked downstream transcriptional effects on angiogenesis and regulatory pathways (Supplemental Figure 7, A, C, and E).

By integrating RNA-Seq, ESR1 ChIP-Seq, and assay for transposase-accessible chromatin using sequencing (ATAC-seq) data, we identified a core set of genes and pathways regulated by activated E2/ESR1 signaling in HAFs in all 3 datasets from *Arom^hum^* LAM (Figure 3C). This contained 58 E2/ESR1-upregulated genes, including well-known E2/ESR1-responsive genes such as *Pgr*, *Pbx1*, and several E2/ESR1-related profibrotic genes (e.g., *Adamts6*, *Fbln7*; Supplemental Table 1). The increase in expression of *Pgr*, a hallmark E2-responsive gene, further demonstrates the successful activation of E2 pathways in HAFs. This elevation was also observed in our prior scRNA-Seq study of *Arom^hum^* LAM (Supplemental Figure 4, A and B) (9). Additionally, *Ltbp1*, an ECM protein involved in TGF-β signaling, was consistently upregulated. The mechanotransduction modulator *Piezo2*, the cell adhesion molecule *Ncam1*, and the semaphorin receptor *Nrp2* were other notable genes upregulated with E2/ESR1 signaling in HAFs (Supplemental Table 1). Functional enrichment analysis of integrated ESR1 ChIP-Seq and RNA-Seq data highlighted the activation of key profibrotic pathways associated with fibroblast proliferation and ECM formation, including WNT, ubiquitin-mediated proteolysis, N-glycan biosynthesis, TGF-β, Hedgehog, and chemokine signaling, in response to E2 treatment (Figure 3D). In contrast, inhibition of E2/ESR1 signaling by fulvestrant uncovered a common set of 34 genes, including cell cycle inhibitors (e.g., Wee1, Cdkn1c, Cdc7) and pathways related to post-transcriptional and translational regulation machinery, as well as phagocytosis and endocytosis (Supplemental Figure 8, A and B, and Supplemental Table 2).

Differential motif analysis from ChIP-Seq and ATAC-seq suggested highly significant enrichment of PLAG1 binding sites adjacent to ESR1 binding sites in distal genomic regions in E2-treated HAFs (Figure 3E). Interestingly, previous research showed that the ectopic expression of PLAG1 in skeletal muscle induces fibrosis and atrophy (22). While we did not observe a significant change in *Plag1* expression with fulvestrant treatment, several other *Plag1*-like genes (*Plagl1*, *Plagl2*, *Plag2l2*) may bind to similar motifs and influence downstream responses. We also identified other enriched regulatory elements unique to E2-treated HAFs, such as NR3C2 (distal region) and HIF1A (promoter region), which were also previously implicated in tissue fibrosis (Figure 3E and Supplemental Figure 8C) (23, 24). Combined network analysis revealed perturbations in pathways associated with cellular and tissue morphogenesis, mesenchymal development, matrisome core, and cell-cell signaling in response to E2 treatment, whereas E2 with fulvestrant led to the enrichment of regulatory transcriptional and developmental pathways (Supplemental Figure 8, D and E). Furthermore, fulvestrant treatment led to an upregulation of apoptotic pathways, as well as heat shock and hypoxic response genes, suggesting that the absence of E2 causes cells to stall in the cell cycle and undergo apoptosis (Supplemental Figure 5D, Supplemental Figure 6C, and Supplemental Figure 8, B and E). Overall, these findings support the notion that E2/ESR1 action at distal genomic regions contributes to the fibrotic pathogenicity of HAFs and that fulvestrant treatment reverses fibrosis by reducing HAF activation and inducing tissue repair pathways.

### Validation of protein and mRNA expression of E2/ESR1 profibrotic genes identified by multiomics analyses of HAFs.

**Figure 4:**
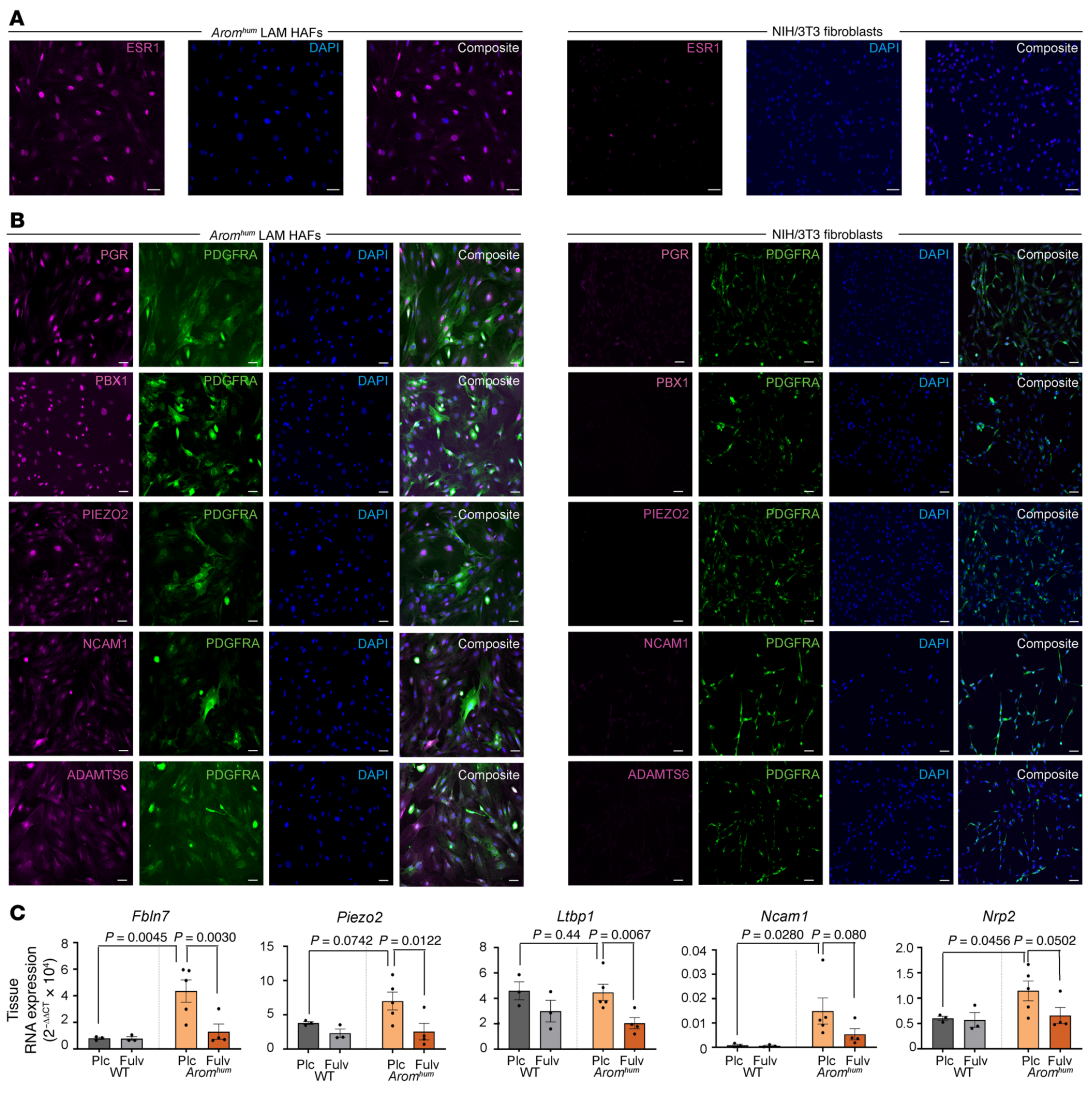
Validation of E2/ESR1-modulated genes in vitro and in vivo. (**A**) In vitro staining of primary cultured HAFs and NIH 3T3 control cells for ESR1. (**B**) PDGFRA- and E2/ESR1-regulated genes identified from multiomics analyses in HAFs and NIH 3T3 cells (*n* = 3–5 mice for HAFs, 3–6 technical replicates; scale bars: 200 μm). (**C**) mRNA expression of the E2/ESR1-targeted genes identified via multiomics analyses of LAMs from *Arom^hum^* mice in the fulvestrant treatment study shown in Figure 2B (*n* = 4–5 per group, mean ± SEM, 2-way ANOVA with Bonferroni multiple comparisons). Plc, placebo; Fulv, fulvestrant.

First, we verified protein expression of key E2/ESR1-responsive genes (*Pgr*, *Pbx1*) and E2/ESR1-related profibrotic genes (*Adamts6*, *Piezo2*, *Ncam1*) identified in multiomics analyses. In E2-treated primary HAFs from *Arom^hum^* mice, we showed expression of PGR, PBX1, ADAMTS6, PIEZO2, and NCAM1 (Figure 4, A and B). Immunofluorescence staining demonstrated coexpression of these proteins with PDGFRA, which can localize in various cellular compartments, including the cell membrane, nucleoplasm, and gap junctions (Figure 4B) (25–28). We also used NIH 3T3 fibroblasts with low ESR1 expression as surrogate controls for fibroblasts from WT mice. As expected, these proteins were either absent or minimally expressed in NIH 3T3 control fibroblasts (Figure 4, A and B). Additionally, we quantified expression of PGR, PIEZO2, CCN3, and PBX1 through flow cytometry following E2 and fulvestrant treatments (Supplemental Figure 4C). Next, we demonstrated significantly increased in vivo mRNA expression of the key E2/ESR1-target genes using the tissues of *Arom^hum^* mice (see Figure 2B), which developed large hernias for 12 weeks (Figure 4C). We verified the upregulation of the core E2/ESR1-related profibrotic genes, including *Fbln7*, *Piezo2*, *Ltbp1*, *Ncam1*, and *Nrp2*, in the LAM of placebo-treated *Arom^hum^* mice. Further, treatment of *Arom^hum^* mice with the E2/ESR1 antagonist fulvestrant significantly decreased LAM mRNA levels of *Fbln7*, *Piezo2*, and *Ltbp1* (Figure 4C). Fulvestrant treatment also decreased expression of *Ncam1* and *Nrp2*, though this did not reach significance (Figure 4C). These in vitro and in vivo data suggest that a critical signature of E2/ESR1-responsive profibrotic genes identified from multiomic genome-wide analyses may be responsible for increased fibroblast proliferation and ECM production, leading to LAM fibrosis and herniation.

**Figure 5:**
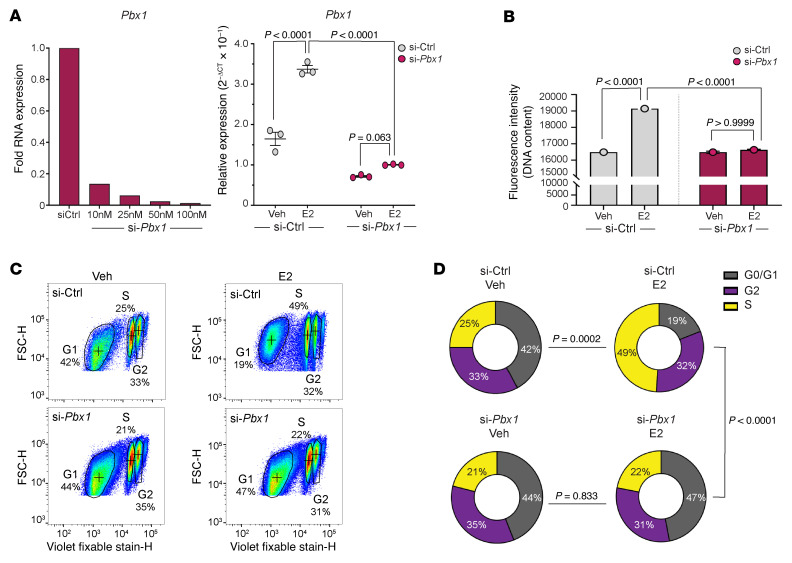
*Pbx1* plays a key role in mediating E2-driven proliferation of HAFs. (**A**) *Pbx1* RNA expression at various siRNA concentrations (left) and following vehicle or E2 treatment (right; 25 nM si-*Pbx1*) (*n* = 3, mean ± SEM, 2-way ANOVA with Bonferroni multiple comparisons). (**B**) DNA content in HAFs treated with vehicle or E2, with and without *Pbx1* knockdown (*n* = 3, mean ± SEM, 2-way ANOVA with Bonferroni comparisons). (**C** and **D**) Flow cytometry scatterplots of HAFs (**C**) and their distribution across cell cycle stages (G0/G1, S, and G2 phases) (**D**) following *Pbx1* knockdown and E2 treatment (*n* = 3 per group, χ^2^ test for proportions).

Given the established role of *Pbx1* as a pioneer factor for ESR1 in breast cancer cells and its necessity for E2 signaling, we conducted siRNA knockdown of Pbx1 to assess its involvement in E2-induced hernia pathogenesis (Figure 5, A and B) (3, 29). HAFs with successful *Pbx1* knockdown exhibited reduced DNA content following E2 treatment as compared with E2-treated control siRNA knockdown HAFs, suggesting an impairment in cell cycle progression (Figure 5B). We further performed flow cytometry to assess the effects of *Pbx1* on the cell cycle. In control siRNA knockdown cells, E2 treatment significantly decreased the percentage of cells in G_0_/G_1_ phase with a concomitant increase in the percentage of cells in S and G_2_ phases (Figure 5C). *Pbx1* knockdown eliminated the effect of E2 in all phases of the cell cycle (Figure 5, C and D). These findings indicate that E2’s proliferative effects are, in part, mediated through *Pbx1*. Similar results were observed with the knockdown of *Ccn3* (Nov), which led to reduced cell proliferation and impacted the downstream production of β-catenin, a known intermediary in *Ccn3* signaling (Supplemental Figure 9) (30).

### E2/ESR1-modulated mRNA or protein expression in Arom^hum^ LAM is comparable to that observed in men with inguinal hernias.

**Figure 6:**
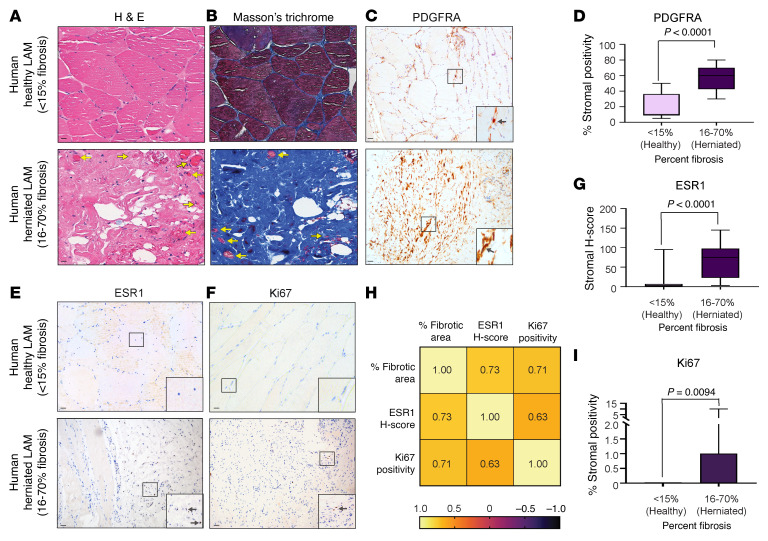
Histopathology of LAM in men with inguinal hernias. (**A**–**C**, **E**, and **F**) Representative images of H&E (**A**), Masson’s trichrome (**B**), and immunohistochemistry staining for PDGFRA (*n* = 25 patients) (**C**), ESR1 (*n* = 34 patients) (**E**), and Ki67 (*n* = 25 patients) (**F**) in human LAM from inguinal hernia sites and adjacent healthy muscle tissues (*t* test; scale bars: 100 μm). Original magnification, ×20 (**C** and **E,** insets). Yellow arrows point to atrophied myofibers, while black arrows point to positive staining. (**D**, **G**, and **I**) Quantification of PDGFRA^+^ (**D**), ESR1^+^ (**G**), and Ki67^+^ (**I**) nuclei from **C**, **E**, and **F**, respectively, stratified by the percentage of fibrosis observed. (**H**) Spearman’s ρ (*r*_s_) correlation between percentage fibrosis, ESR1, and Ki67 scores (44 samples from 22 patients, median ± interquartile range).

We analyzed LAM from men undergoing hernia surgery to examine E2/ESR1-mediated mRNA and protein expression and associated histological changes in human inguinal hernias. We collected matched biopsies from the herniated LAM and adjacent healthy-appearing LAM from 25 men undergoing hernia repair surgery (21–76 years of age). The adjacent healthy-appearing tissue exhibited lower levels of fibrosis (<15%), consistent with our previous findings in LAM tissues from nonhernia patients (Supplemental Figure 10A) (6). Moreover, this 15% threshold aligns with the collagen levels observed in WT mice without herniation, providing a meaningful baseline (Figure 1E). We observed extensive muscle fibrosis containing atrophic myofibers in human herniated LAM by Masson’s trichrome staining, with fibrosis ranging from 5% to 70% (Figure 6, A and B). Immunoreactive PDGFRA was found only in stromal fibroblasts but not myofibers (Figure 6C). Expression of ESR1 and the cell proliferation marker Ki67 were observed in a strikingly higher number of cells in hernia site LAM compared with adjacent healthy muscle (Figure 6, E and F). Expression of PDGFRA, ESR1, and Ki67 was significantly higher in LAM from herniated samples (16%–70%) compared with healthier tissues (<15%; Figure 6, D, G, and I). Moreover, we observed a significant correlation between the expression of ESR1 and Ki67 and the degree of fibrosis in herniated LAM (Figure 6H).

**Figure 7:**
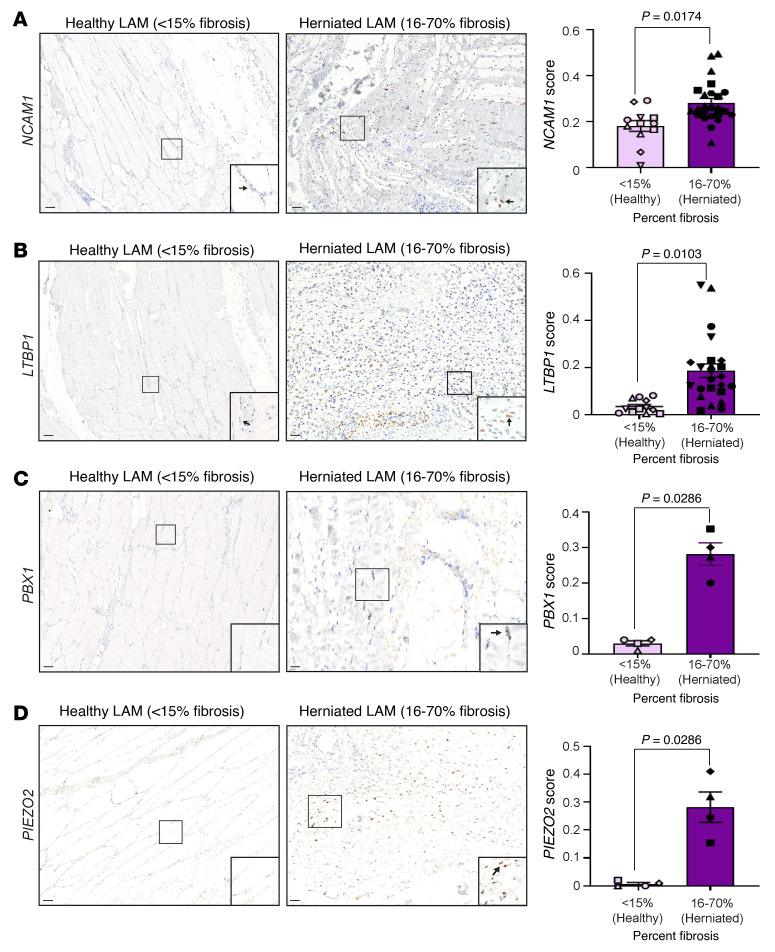
E2/ESR1-modulated genes in men with inguinal hernias. Representative RNAscope images of the genes *NCAM1* (**A**) and *LTBP1* (**B**) identified from multiomics studies that were observed in all patient samples and their quantification, stratified by the size of the fibrotic area (*n* = 12 tissues from 6 patients denoted by different shapes, mean ± SEM; scale bars: 200 μm). RNAscope images of *PBX1* (**C**) and *PIEZO2* (**D**) identified from multiomics studies that were observed in some patient samples. Black arrows point to positive staining (*n* = 8 tissues from 4–5 patients denoted by different shapes, mean ± SEM, nested *t* test; scale bars: 200 μm). Original magnification, ×40 (**A**–**D**, insets).

To gain deeper molecular insights, we used RNA in situ hybridization and verified the expression of E2/ESR1-modulated genes identified in *Arom^hum^* LAM (e.g., *NCAM1*, *LTBP1*, *ADAMTS6*, *NRP2*, *PBX1*, and *PIEZO2*) in the fibrotic regions of herniated muscle tissue from men (Figure 7, A–D, and Supplemental Figure 10, B–D). Moreover, both *NCAM1* and *LTBP1* were consistently expressed in all patient samples, providing evidence of their involvement in the hernia development (Figure 7, A and B). Additionally, we detected PGR protein expression via immunohistochemistry (Supplemental Figure 10D) and mRNA expression of *ADAMTS6*, *NRP2*, *PBX1*, and *PIEZO2* in more than 30% of the herniated and fibrotic LAM samples (Figure 7, C and D, and Supplemental Figure 10, B and C). Overall, our findings demonstrate that the activation of E2/ESR1 signaling in LAM fibroblasts from a large subset of men with inguinal hernias is similar to that observed in HAFs from *Arom^hum^* mice, emphasizing the clinical relevance of E2/ESR1 signaling in inguinal hernias in men.

## Discussion

The LAM, composed of transverse, internal, and external oblique muscles, as well as a vast stromal network, plays a crucial role in maintaining abdominal integrity (31). Disturbances in collagen and ECM proteins and genetic variations have been associated previously with hernia development (32–37). Our study adds to these findings by demonstrating that E2/ESR1 induces profibrotic genes and pathways that can propagate hernia pathology. Furthermore, our use of the *Arom^hum^* model, which mimics the elevated E2 levels characteristic of aging men — a demographic disproportionately affected by inguinal hernias — enhances the clinical relevance and significance of our findings (3).

We were intrigued that the administration of fulvestrant to adult mice with well-established large scrotal hernias led to complete regression of fibrosis, return of myofiber size to normal, and restoration of normal anatomy with spontaneous reduction of the hernias, since this is unprecedented (Figure 2). The use of PDGFRA-cre mice facilitated the selective ablation of ESR1 in PDGFRA^+^ FAPs, underscoring the sufficiency of these cells in the fibrotic processes associated with hernias (Figure 1). While several studies suggested approaches for preventing or alleviating fibrosis (17–19), there are no published interventions that achieved complete reversal of skeletal muscle fibrosis. We demonstrate that pharmaceutical targeting of E2/ESR1 signaling reverses fibrosis in adult mice and provides insights into potential skeletal muscle regeneration mechanisms.

The multiomics approach identified a core set of profibrotic genes regulated by E2/ESR1, providing deeper mechanistic insights (Figure 3 and Supplemental Table 1). We revealed E2/ESR1-induced *ADAMTS6* and its substrate *LTBP1*, possibly triggering downstream TGF-β signaling upon cleavage (38). To support prior hypotheses that estrogen-mediated mechanotransduction is controlled through PIEZO1/2 in the skeletal system, we present evidence for the induction of the *PIEZO2* channel via E2/ESR1 activation (39). Polymorphisms and dysregulation of ECM-related fibulins have previously been linked to inguinal hernia susceptibility (40–43). In our study, the direct induction of both *FBLN7* and *FBLN3* by E2/ESR1 amplifies the potential applicability of these findings across a broad spectrum of inguinal hernias. Our findings suggest that E2-activated ESR1 serves as a master regulator of a broad signature of genes instrumental for skeletal muscle fibroblast proliferation, ECM formation, and myocyte atrophy in lower abdominal musculature.

Moreover, E2 treatment significantly induced other TGF-β pathway genes, such as *Smad3* and *Tgfb2*, which play critical roles in fibroblast biology and fibrogenesis (Supplemental Figure 7B) (44). This selective upregulation of the TGF-β pathway mimics the increased proliferation seen in an acute injury response, whereas inhibiting E2/ESR1 signaling via fulvestrant suppresses this mechanism, promoting hernia regression and regeneration pathways (Figure 3D and Supplemental Figure 3). The E2-induced fibrotic pathways and ECM deposition observed in our study align with observations in various other TGF-β–driven conditions such as dermal fibrosis, systemic sclerosis, benign prostatic hyperplasia, and hepatic fibrosis, underscoring the potential widespread relevance of our findings (45–49). Conversely, fulvestrant treatment increased pathways related to TNF-α, hypoxia, oxidative stress, and cell cycle regulation, suggesting apoptosis of pathogenic fibroblasts (Supplemental Figure 5D, Supplemental Figure 6C, Supplemental Figure 7C, and Supplemental Figure 8B). The observed reduction in fibrosis alongside muscle regeneration points to possible cell-cell interactions between HAFs and muscle progenitors, which could be a promising area for future research.

Our multiomics analysis primarily focused on comparing the effects of the E2 group and the E2 plus fulvestrant group. This approach aimed to replicate both E2-replete and E2-depleted conditions to better understand the effects of E2 signaling in HAFs. However, we aimed to increase the robustness of our analysis by validating our findings in vivo using mouse and human LAM. Although our study highlights the role of ESR1 in reversing fibrosis through pharmacological interventions, it does not rule out the involvement of ESR1 in nonfibroblast cells or its nongenomic actions. Future investigations could incorporate temporal genetic manipulation of fibroblast-specific ESR1 to better understand its role in tissue reversibility. These approaches would offer a more comprehensive view of the mechanisms involved and add to our findings. Moreover, the lack of data on the sex steroid profile of the human samples represents another limitation. Future studies should include measurements of E2, aromatase, and other serum- or tissue-level hormones to explore potential correlations with the observed fibrotic changes. Additionally, employing single-cell techniques or multiplexing to measure multiple genes in fibroblasts could provide more detailed insights into the molecular mechanisms of fibrosis and herniation. Only male *Arom^hum^* mice develop scrotal hernias, though both sexes express ESR1 in the LAM. The absence of similar phenotypic changes in female mice may be attributed to sex-specific effects of ESR1 and anatomical differences. This observation parallels human studies, where 97% of inguinal hernias occur in males (1). Although estrogen effects on various muscles and fibroblasts have been studied, there is limited understanding of its role in LAM. Further research is needed to explore estrogen’s effects on female abdominal muscles, particularly regarding changes during pregnancy.

Although estrogen excess has been reported to be associated with fibrosis-related pathologies of the breast, testes, liver, and lung, the underlying cellular and molecular mechanisms remained unknown (50–54). In the lung, elevated estrogen is linked to fibrotic conditions like pulmonary arterial hypertension and lymphangioleiomyomatosis, with a higher prevalence of such diseases in women (39, 55, 56). Conversely, estrogen appears to be protective against liver fibrosis, as indicated by its negative correlation with hepatic stellate cell activation and TGF-β expression (57, 58). Women with higher estrogen levels typically show slower liver fibrosis progression. Estrogen’s role in kidney fibrosis is similarly complex: it regulates oxidative stress and fibrosis in chronic kidney disease but has mixed effects in autoimmune disorders (59, 60). This report establishes the estrogen receptor ESR1 expressed in a unique fibroblast population as the key mechanistic mediator of estrogen-driven fibrosis, which is reversible via gene knockout or selective pharmaceutical intervention. Our in vivo and in vitro studies have provided valuable insights into the intricate molecular processes driving skeletal muscle fibrosis and offer promising avenues for developing targeted interventions for inguinal hernias, particularly in high-risk older populations, and other fibrotic diseases.

## Methods

Further information can be found in Supplemental Methods.

### Sex as a biological variable

Our study was carried out in male mice and tissues from human males. Inguinal hernia affects predominantly men compared with women (10:1 ratio). No female *Arom^hum^* mice developed hernias.

### Mouse experiments

#### Arom^hum^ mouse model.

Bacterial artificial chromosome (BAC) DNA with aromatase gene coding regions (exon II and exon X) and promoter regions (I.1, I.4, I.7, I.f, I.6, I.3, and PII) was created and injected into FVB/N-fertilized oocytes (Genetically Engineered Mouse Core, Baylor College of Medicine, Houston, Texas, USA) (5, 6, 9). Mice were maintained on a 14-hour light/10-hour dark cycle with standard chow (Envigo, Teklad LM-485, 7912 for nonbreeders, S-2335 7904 for breeders). Genotyping was performed according to previous studies (6). Scrotal dimensions were measured using a digital caliper [area (mm^2^) = length (mm) × width (mm)] in the morning 2–3 times a week by a single experimenter to reduce variability. All endpoint tissue collections were performed before 1 pm to avoid hormone fluctuations. Mice were euthanized with ketamine-xylazine (100 mg/kg, 10 mg/kg) followed by cervical dislocation. LAM tissue (the lower third of the abdominal muscle) was harvested as described previously (6).

#### fEsr1^–/–^ Arom^hum^ mouse model.

C57BL/6 *Pdgfra-cre* mice (The Jackson Laboratory 013148) were crossed with B6 *Esr1*-flox (The Jackson Laboratory 032173) to generate fibroblast-specific ESR1-knockout mice (*fEsr1^–/–^*). These mice were then crossed with *Arom^hum^* mice to generate mice expressing aromatase without ESR1 expression in LAM fibroblasts (*fEsr1^–/–^ Arom^hum^*). *fEsr1^+/+^ Arom^hum^* mice were used as controls.

#### Genotyping primers.

The following genotyping primers were used: *Arom^hum^* forward, AGTATCCCGGTGGAGTGATCT; *Arom^hum^* reverse, AAGCTGGCTGAAAGTCTAGGG; *Pdgfra*-cre forward, TCAGCCTTAAGCTGGGACAT; *Pdgfra*-cre reverse, ATGTTTAGCTGGCCCAAATG; *Esr1*-flox P1, TTGCCCGATAACAATAACAT; *Esr1*-flox P2, ATTGTCTCTTTCTGACAC; *Esr1*-flox P3, GGCATTACCACTTCTCCTGGGAGTCT.

#### Subcutaneous pellet implantation.

Mice were anesthetized with 1%–3% inhalational isoflurane or intraperitoneal ketamine-xylazine (100 mg/kg, 10 mg/kg). After fur removal, slow-release drug pellets were inserted into an incision on the skin by the neck of the mouse and sealed via wound clips. One milligram per kilogram body weight of meloxicam was administered as an analgesic after surgery. For prevention studies, pellets were inserted in 3- to 4-week-old mice. For treatment studies, pellets were inserted once large hernias were formed (~200 mm^2^, ~6–10 weeks old). Mice were monitored and hernias were measured 2–3 times a week for 12 weeks. Custom fulvestrant (3.75 mg/pellet; Sigma-Aldrich 1286650), raloxifene HCl (4.05 mg/pellet; Sigma-Aldrich R1402), MPP (2.25 mg/pellet; Sigma-Aldrich M7068), PHTPP (1.8 mg/pellet; Sigma-Aldrich SML1355), and G-15 (0.9 mg/pellet; Cayman Chemical Co. 14673) pellets were produced by Innovative Research of America (X-999; placebo C-111) (61–63).

### Human LAM samples

The human study was approved by the Institutional Review Boards of Northwestern University and the University of Texas Health Science Center at Houston, and informed consent was obtained from all patients before hernia surgery performed at the University of Texas Health Science Center at Houston (STU00208860). Two biopsy specimens were obtained from each patient (1 × 0.5 × 0.5 cm^3^ for each biopsy), one from the hernia site and another from adjacent healthy-appearing muscle. Thirty-five samples were obtained (34 men and 1 transgender woman). None of the patients were receiving hormone therapy when the tissues were collected. The average age was 51.06 years (SD ±14.59 years, range 21–76 years). The average weight and height at time of surgery were 81.84 kg (SD ±11.73 kg) and 66.05 in. (SD ±6.96 in.), respectively, with an average BMI of 30.39 kg/m^2^ (SD ±11.17). 44.8% of patients underwent right inguinal hernia surgery, and 55.2% underwent left inguinal hernia surgery.

### In vitro HAF experiments

#### Fibroblast isolation.

Fibroblasts were isolated as previously described (64). LAMs from mice were harvested and placed in wash medium (Hyclone Ham’s F-10 nutrient mixture with 1 mM l-glutamate [GE Life Sciences], 10% horse serum [Life Technologies], and penicillin-streptomycin [Gibco]) on ice. LAMs were minced into a slurry and incubated in muscle dissociation buffer (wash medium plus 1,000 U/mL collagenase II [Worthington Biochemical]) at 37°C with 70 rpm agitation for 1 hour. The cells were then washed and resuspended in fibroblast growth medium (Ham’s F-12 medium with 10% FBS [Gibco], penicillin-streptomycin [Gibco], and Plasmocin prophylactic [InvivoGen]) and grown in 0.2% gelatin–coated tissue culture plates. After 1 hour, LAM fibroblasts adhered to the plate, and the supernatant containing other cell types was removed. The adherent HAFs were grown to 80%–90% confluence before passaging. One hundred percent of isolated LAM fibroblasts were positive for both ESR1 and PDGFRA, indicating that HAFs were obtained using this protocol.

#### Estrogen and fulvestrant treatments.

HAFs were passaged once (P1) and grown to approximately 70% confluence. Cells were starved overnight (~16 hours) in phenol red–free, serum-free medium (Ham’s F-12 without phenol red with penicillin-streptomycin [Gibco] and Plasmocin prophylactic [InvivoGen]). For ChIP-Seq and ATAC-seq, all cultures were in serum-free Ham’s F-12 medium; for RNA-Seq, 0.1% charcoal-stripped FBS (Gibco) was added to ensure cell survival during the longer incubation time (48 hours). HAFs were pretreated with 100 nM fulvestrant or DMSO for 3 hours. Ten-nanomolar E2 or ethanol was subsequently added. After 1 hour, HAFs were harvested for ChIP-Seq and ATAC-seq (65–67). For RNA-Seq, cells were incubated in estrogen-replete (E2) or estrogen-inhibited (E2 plus fulvestrant) conditions for 48 hours.

#### siRNA knockdown treatments.

HAFs were grown to approximately 70% confluence and were starved overnight (~16 hours) in phenol red–free, serum-free medium (Ham’s F-12 without phenol red with penicillin-streptomycin [Gibco] and Plasmocin prophylactic [InvivoGen]). 25 nM siRNA targeting *Pbx1* and *Ccn3* or negative controls was added during starvation stage (Horizon Discovery, L-042709-00-0005, L-040684-01-0005). 10 nM E2 or ethanol was subsequently added in 0.1% charcoal-stripped FBS (Gibco). HAFs were lysed and RNA was extracted using an RNeasy Mini Kit (QIAGEN 74104) according to the manufacturer’s instructions. Knockdown efficiency was confirmed through quantitative PCR (qPCR) (Thermo Fisher Scientific TaqMan Gene Expression Assays 4351372; *Ccn3*: Mm00456855_m1; *Pbx1*: Mm04207622_m1). *Pbx1* mouse accessions are as follows: NM_001291508, NM_001291509, NM_008783, NM_183355, XM_006496699, and XM_006496700; the mouse accession for *Ccn3* is NM_010930.

### RNA sequencing

#### RNA extraction.

HAFs were lysed and RNA was extracted using an RNeasy Mini Kit (QIAGEN 74104) according to the manufacturer’s instructions. Briefly, cells were lysed using RLT buffer, mixed with an equal volume of 70% ethanol, and transferred to spin columns. After 2 washes with RW1 and RPE, RNA was eluted in PCR-grade water. RNA was quantified using a Qubit Fluorometer (Invitrogen), and integrity was assessed using a 2100 Bioanalyzer (Agilent Technologies). All samples had an RNA integrity number greater than 9.

#### Library preparation and sequencing.

Libraries were prepared from 1 μg of sample RNA using the KAPA RNA HyperPrep Kit with RiboErase (HMR, Roche 08098131702) according to the manufacturer’s instructions and sequenced on the Illumina NovaSeq 6000 platform (paired-end, 150 bp, 100 million reads).

#### Data processing and quality control.

All arguments were set to default values unless specified below. FASTQ files were quality-checked using FastQC v0.11.5 (68). All files passed quality checks on per-base sequence quality, per-base GC content, per-base N content, and sequence length distribution. Adapters were trimmed using Trimmomatic v0.39, and SortMeRNA v2.1 was used to remove any traces of rRNA (69, 70). Files were aligned to mm10 genome using STAR v2.5.2 with *--outFilterScoreMinOverLread* and --*outFilterMatchNminOverLread* values set to 0.5. Samtools v1.14 was used to sort BAM files (71, 72). HTSeq v0.13.5 (*htseq-count* with *–s reverse*) was used to count reads (73). Subsequent data analysis was performed in R v4.1.1.

### ESR1 ChIP and ATAC sequencing

#### ChIP library preparation and sequencing.

Cells were fixed in paraformaldehyde for 15 minutes at room temperature. Processing and library preparation were performed by Active Motif Services. Thirty micrograms of chromatin was used with 4 μg of ESR1 antibody (MilliporeSigma 06-935). One negative control primer (*Untr6*) and 2 positive control primers (*Pgr* and *Greb1*) were used for ChIP-qPCR. Enrichments of positive control signals over background were between 4- and 27-fold, indicating good chromatin quality and successful E2 treatment. Sequencing was performed using the Illumina NextSeq 500 platform (single-end, 75 bp).

#### ATAC sample, library preparation, and sequencing.

Cell pellets were processed according to the manufacturer’s instructions using the ATAC-Seq Kit (Active Motif 53150). In brief, DNA was tagmented and amplified using i7 and i5 primer combinations for 10 PCR cycles. The DNA was purified and quality-checked using a 2100 Bioanalyzer (Agilent Technologies). Double-sided selection was performed, and the libraries were sequenced on the Illumina NovaSeq 6000 platform (paired-end, 150 bp, 100 million reads).

#### Data processing and quality control.

Arguments were set to default values unless specified below. All FASTQ files passed quality checks (per-base sequence quality, per-base GC content, per-base N content, sequence length distribution) via FastQC v0.11.5 (68). Reads were subsequently trimmed using Trimmomatic v0.39 (69). Sequences were aligned to mm10 genome using Bowtie2 aligner v2.4.2 (--*very-sensitive*) (74). Samtools v1.14 was used to convert SAM to BAM, and duplicates were removed using Picard v2.21.4 (*REMOVE_DUPLICATES* = *true, REMOVE_SEQUENCING_DUPLICATES* = *true*) (72, 75). After mitochondrial reads were removed (*sed ‘/chrM/d;/random/d;/chrUn/d’*), ChIP-Seq peak calling was performed using MACS2 v2.2.71 (*callpeak* with *--keep-dup all*) (76, 77). For ATAC, peak calling was performed using v2.2.71 (*callpeak* with *--nomodel --shift -100 --extsize 200 --keep-dup all*) (76, 77). HOMER v4.10 was used to discover motif enrichment (78). Subsequent analysis was performed using R v4.1.1.

### Data analysis of multiomics sequencing data sets

#### RNA-Seq.

Count tables were fed into the DESeq2 package v1.37 to identify differentially expressed genes (79). Counts fewer than 10 were removed from analysis, and normalization was performed via a median of ratios method. Since the principal component analysis (PCA) plot (*plotPCA*) showed batch variations, SVA (*ComBat_seq*) and Limma packages (*limma: removeBatchEffect*) were used to reduce batch effects (80, 81). AnnotationDbi, GenomicRanges, and org.Mm.eg.db were used for annotation. A heatmap of DESeq2 (adjusted *P* value [*P*_adj_] < 0.05, fold change > 1.2) results was generated using the pheatmap package (82–84). Gene set enrichment analysis was performed by WebGestalt using differentially expressed genes (*P*_adj_ < 0.05) identified via DESeq2 results (85, 86).

#### ChIP-Seq and ATAC-seq.

ChIPQC v1.30.0 reports of all samples exhibited good enrichment of peaks (0% duplicates, relative cross-coverage score between 1.5 and 4, reads overlapping in blacklisted regions [RiBL] 1%–2.5%, fragment length cross coverage ~200, mean read length ~75, and strong signal in cross-correlation plots) (87). To reduce RiBL scores, blacklisted peaks (ENCODE file: ENCFF999QPV) were filtered out. The ChIPseeker package was used to annotate peaks to *TxDb.Mmusculus.UCSC.mm10.knownGene* (88). Consensus peaks were counted, and differentially expressed peaks were identified using DESeq2 (*P*_adj_ < 0.05) (79). Limma was used to remove batch effects (*limma: removeBatchEffect*) (81). A heatmap was generated using pheatmap (89). Common peaks across replicates were exported to BED files to BED files and visualized on Integrative Genome Viewer (Broad Institute) (90). Pathway analysis was performed using DAVID function Gene Ontology (GO) clustering (91). Unique peaks were separated into distal and promoter regions before proceeding to motif enrichment via HOMER v4.11 (*findMotifsGenome.pl*, *-size* 200, *P*_adj_ < 0.05) (78).

#### Integration.

Upregulated genes in samples treated with E2 or E2 plus fulvestrant with a fold change of 1.2 or greater from all 3 datasets were combined to identify common genes. HOMER (*findMotifsGenome.pl, -size* 200, *P*_adj_ < 0.05) was used to find enriched motifs in distal and promoter regions (78). Kyoto Encyclopedia of Genes and Genomes pathway enrichment analysis was performed by CistromeGO (92). Network analysis was performed using Cytoscape v3.9.1 with shared genes from at least 2 of the 3 datasets using GO enrichment from EnrichR and ReactomeFI plug-ins (93–96).

### Flow cytometry

Single-cell suspensions of LAM were obtained as described above. Cell suspensions passed through a 70 μm cell strainer were used for flow cytometry staining. Cells were first stained with a live/dead fixable stain (Invitrogen L34961) for 30 minutes at room temperature in the dark. After washing, cells were subsequently stained with PDGFRA antibody (Invitrogen 11-1401-82) for 30 minutes on ice in the dark. Next, cells were fixed with Foxp3 Transcription Factor Staining Buffer Set (Invitrogen kit 00-5523-00) according to the manufacturer’s instructions. ESR1 (MilliporeSigma 06-935), PBX1 (Invitrogen PA517223), PIEZO2 (Invitrogen PA572976), PGR (ABclonal A0321), or CCN3 (Cell Signaling Technology 8767S) was added, and cells were incubated for 1 hour on ice in the dark, followed by 2 washes. Secondary antibodies (Invitrogen 12-4739-81 and 62-4137-82) were added for 1 hour on ice before flow cytometry was performed. For cell cycle analysis, HAFs were treated with FxCycle Stain (Thermo Fisher Scientific F10347) alone. Samples were run on the BD LSRFortessa SORP 6-laser Cell Analyzer. Data analysis was performed using FlowJo v10.6.2 software. For analysis, single cells were separated out using forward and side scatter plots. Dead cells were removed and gating for other parameters was set based on single-color controls and fluorescence-minus-one controls.

### Statistics

Analysis of RNA-Seq, ChIP-Seq, and ATAC-seq data was performed as described above. For other comparisons, the data were first checked for normality using the Shapiro-Wilk test or via Q-Q plots. If normality was not met, comparable nonparametric tests (Kruskal-Wallis, Wilcoxon’s signed rank, Spearman’s correlation) were performed. Two-way ANOVA was performed for experiments with more than 2 groups. Post hoc multiple pairwise comparisons were corrected for multiple comparisons using the Dunn-Bonferroni method (97). For comparisons between 2 groups, 2-tailed *t* tests were performed, and χ^2^ test was used to compare cell cycle proportions. A *P* value less than 0.05 was considered significant.

### Study approval

Animal experimental protocols were approved by the Institutional Animal Care and Use Committee at Northwestern University. The human study was approved by the Institutional Review Boards of Northwestern University and the University of Texas Health Science Center at Houston.

### Data availability

FASTQ and processed files of the sequencing data were deposited in the NCBI’s Gene Expression Omnibus (GEO) database GSE226868 super series — GSE226859 (ChIP-seq), GSE226860 (ATAC-seq), and GSE226867 (RNA-Seq).

## Author contributions

TP performed data analysis and visualizations and wrote the original draft of the article. TP, DJE, and TY performed investigation. SEB, HZ, PY, JJS, and YD conceptualized and supervised the study. SEB, RLL, and HZ acquired funding. All authors established methodology and reviewed and edited the manuscript.

## Supplementary Material

Supplemental data

Supporting data values

## Version 1 02/04/2025

In-Press Preview

## Version 2 03/17/2025

Electronic publication
