# Supplementary material for: Estrogen receptor-α ablation reverses muscle fibrosis and inguinal hernias
Source: J Clin Invest. 2025 Feb 4;135(6):e179137. doi: 10.1172/JCI179137 (PMC11910215; doi:10.1172/JCI179137)
Supplement: Supplemental data [file jci-135-179137-s131.pdf]

Estrogen Receptor Alpha Ablation Reverses Skeletal Muscle Fibrosis and Inguinal Hernias

Authors:

Tanvi Potluri[1], Tianming You[1], Ping Yin[1], John Coon V[1], Jonah J. Stulberg[2], Yang Dai[3], David J. Escobar[4], Richard L. Lieber[5-7], Hong Zhao[1†*] and Serdar E. Bulun[1†*]

Affiliations:

[1]Department of Obstetrics & Gynecology, Feinberg School of Medicine, Northwestern University, Chicago, USA
[2]Department of Surgery, McGovern Medical School at the University of Texas Health Sciences Center, Houston, USA
[3]Department of Biomedical Engineering, University of Illinois at Chicago, Chicago, USA
[4]Department of Pathology, Feinberg School of Medicine, Northwestern University, Chicago, USA
[5]Departments of Physical Medicine and Rehabilitation and Biomedical Engineering, Northwestern University, Chicago, USA
[6]Research Service, Hines VA Medical Center, Maywood, IL USA
[7]Shirley Ryan AbilityLab, Chicago, USA
[†]These authors contributed equally to this work

The authors have declared that no conflict of interest exists

*Corresponding authors:

Serdar E. Bulun, M.D.

Department of Obstetrics & Gynecology,

Feinberg School of Medicine,

Northwestern University, Chicago, 60657, USA

Phone: +1 312.472.3980

Email: s-bulun@northwestern.edu

Hong Zhao, MD, PhD

Department of Obstetrics & Gynecology,

Feinberg School of Medicine,

Northwestern University, Chicago, 60657 USA

Phone: +1 312.503.0780

Email: h-zhao@northwestern.edu

SUPPLEMENTAL FIGURES

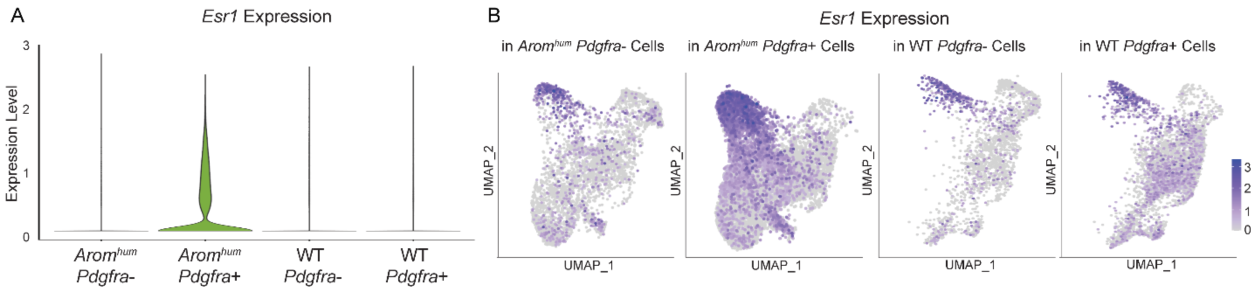

Supplementary Figure S1: *Esr1* and *Pdgfra* expression in LAM fibroblasts

(A) Violin plots of *Esr1* expression from a previously published single-cell RNAseq dataset in *Pdgfra*+ and *Pdgfra*- fibroblast cells in WT and *Arom*^{hum} mice. **(B)** Feature plots of *Esr1* expression from a previously published single-cell RNAseq dataset in *Pdgfra*+ and *Pdgfra*- fibroblast cells in WT and *Arom*^{hum} mice (n = 3) (1).

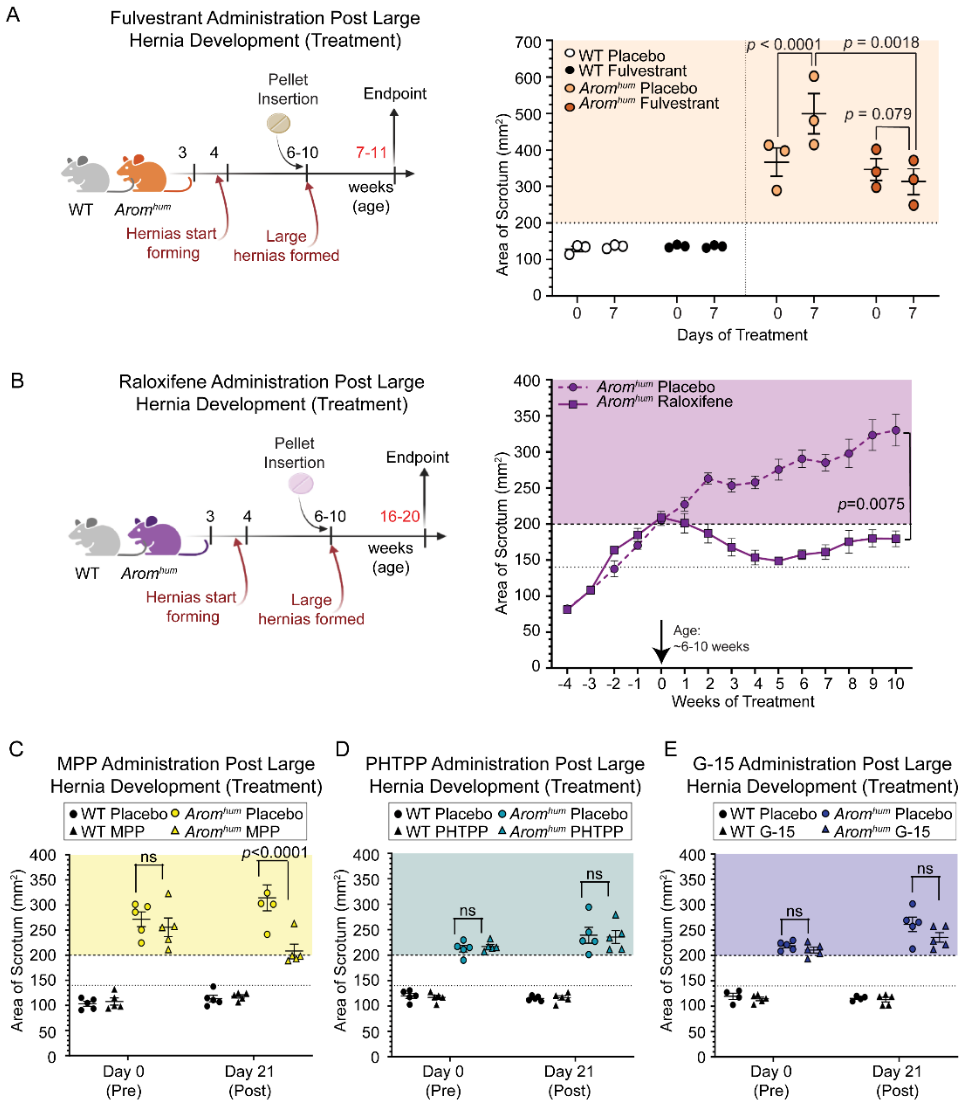

Supplementary Fig. S2: Inhibition of E2/ESR1 signaling in *Arom^hum* mice with large hernias

(A) Schematic of short 7-day fulvestrant treatment study design (left) and measurement of scrotal hernias (right); fulvestrant was administered after severe and large hernias formed for 7 days. (n = 3/group, mean ± S.E.M, ANOVA). **(B)** Schematic of raloxifene treatment study design (left) and measurement of scrotal hernias (right); raloxifene was administered after large hernias formed. Arrow indicates the week of pellet implantation (n = 7-10/group, mean ± S.E.M, repeated measure ANOVA). Measurement of scrotal

hernias prior to and post **(C)** MPP, **(D)** PHTPP, and **(E)** G-15 administration (n = 4-5/group, mean ± S.E.M, ANOVA). In (A and B), the dotted lines at 140 mm^2 represents normal scrotum size prior to hernia development, and the shaded regions in (A) – (D) represents large scrotal hernia size (>200 mm^2). ns = not significant.

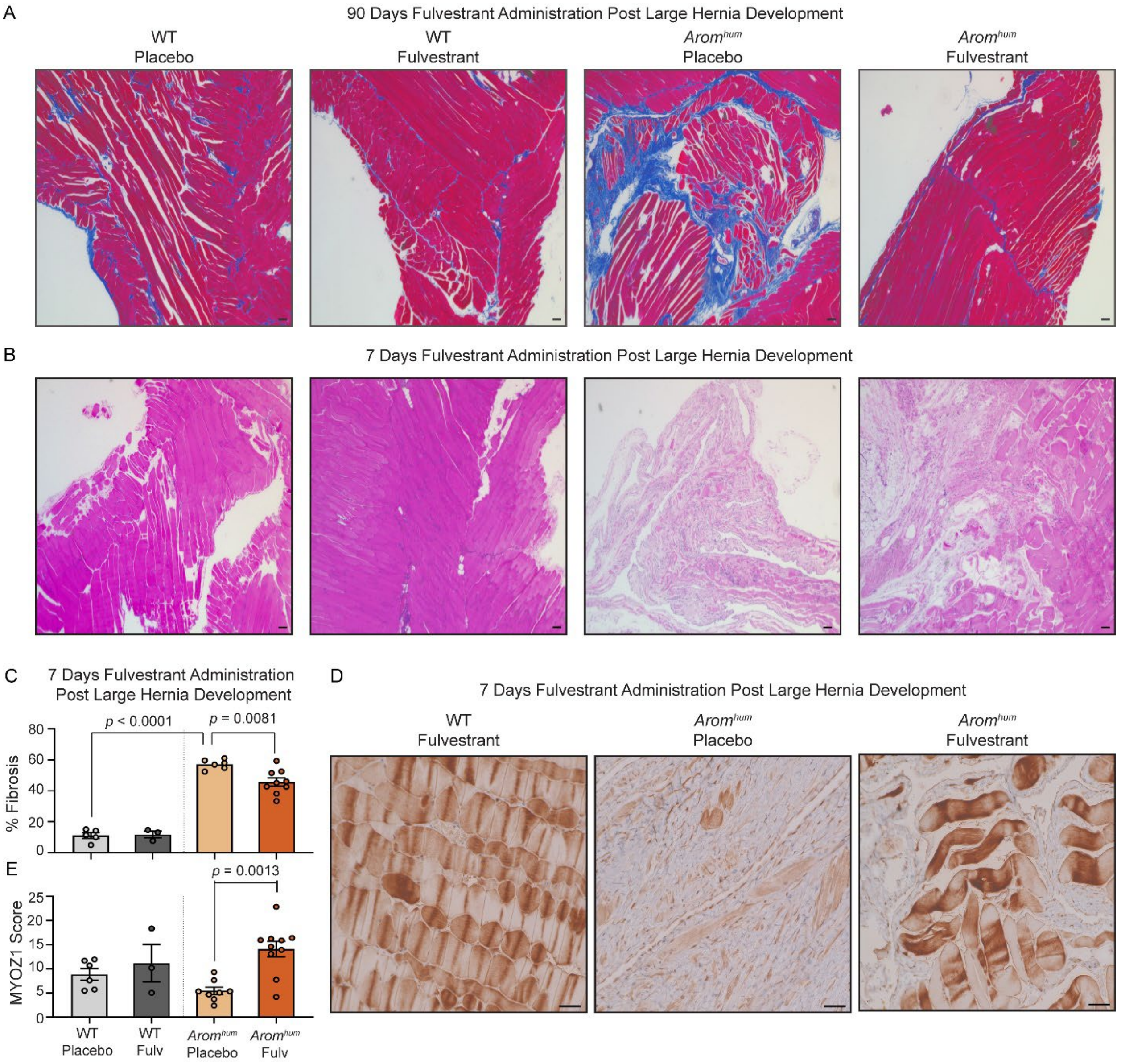

Supplementary Fig. S3: Histopathology of LAM post fulvestrant administration

Representative images of LAM of mice after a **(A)** 90-day fulvestrant treatment from Figure 2B and **(B)** 7-day fulvestrant treatment from Figure S2A. **(C)** Quantification of percent fibrosis post 7-day fulvestrant treatment. **(D)** Immunohistochemistry of MYOZ1 and its **(E)** quantification in mice treated with fulvestrant for 7 days (n = 3 mice/group mean ± S.E.M, ANOVA with t-test, Scale bars, 100 µm)

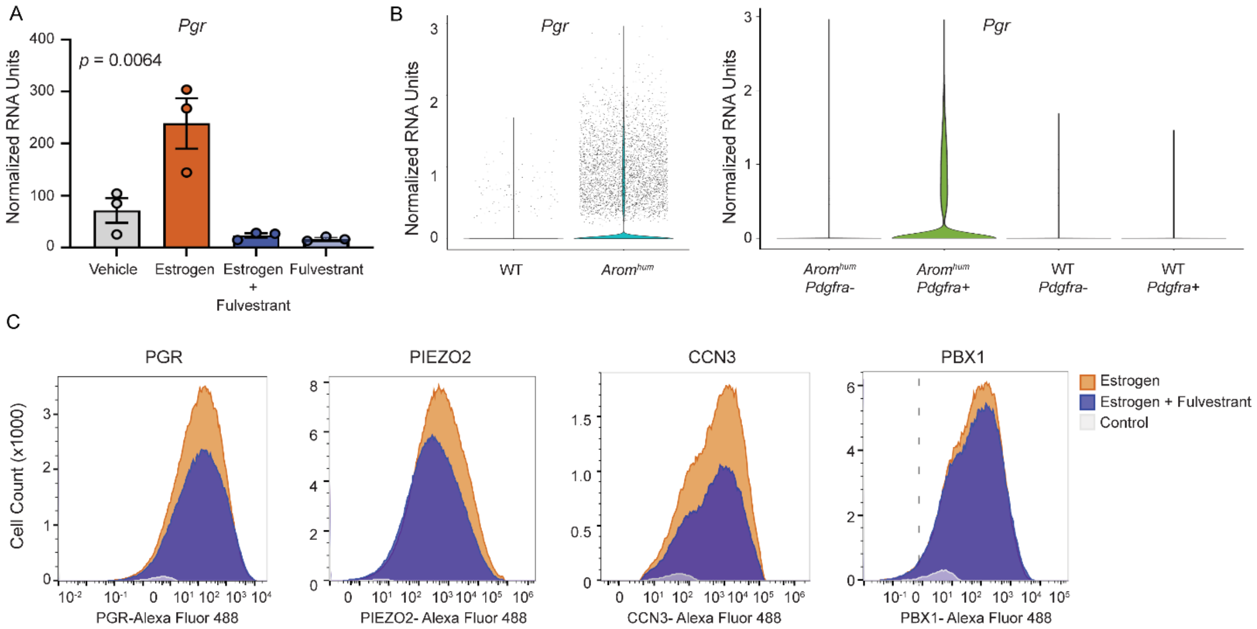

Supplementary Fig. S4: Expression of key genes in HAFs

(A) Pgr, a downstream gene indicative of E2 signaling, and its expression in HAFs treated with vehicle (ethanol, DMSO), E2, E2 plus fulvestrant or fulvestrant alone (n = 3, mean ± S.E.M, ANOVA). **(B)** *In vivo* Pgr expression in WT and *Arom^hum^* LAM in a previously published sc-RNAseq dataset (left). Violin plots of Pgr expression in fibroblasts of WT and *Arom^hum^* LAM, stratified by co-expression of *Pdgfra* (right) (n = 3). **(C)** Flow cytometric quantification of key E2-responsive genes (PGR, PIEZO2, CCN3, PBX1) in HAFs treated with E2 or E2 with fulvestrant (n = 3-5 mice/group)

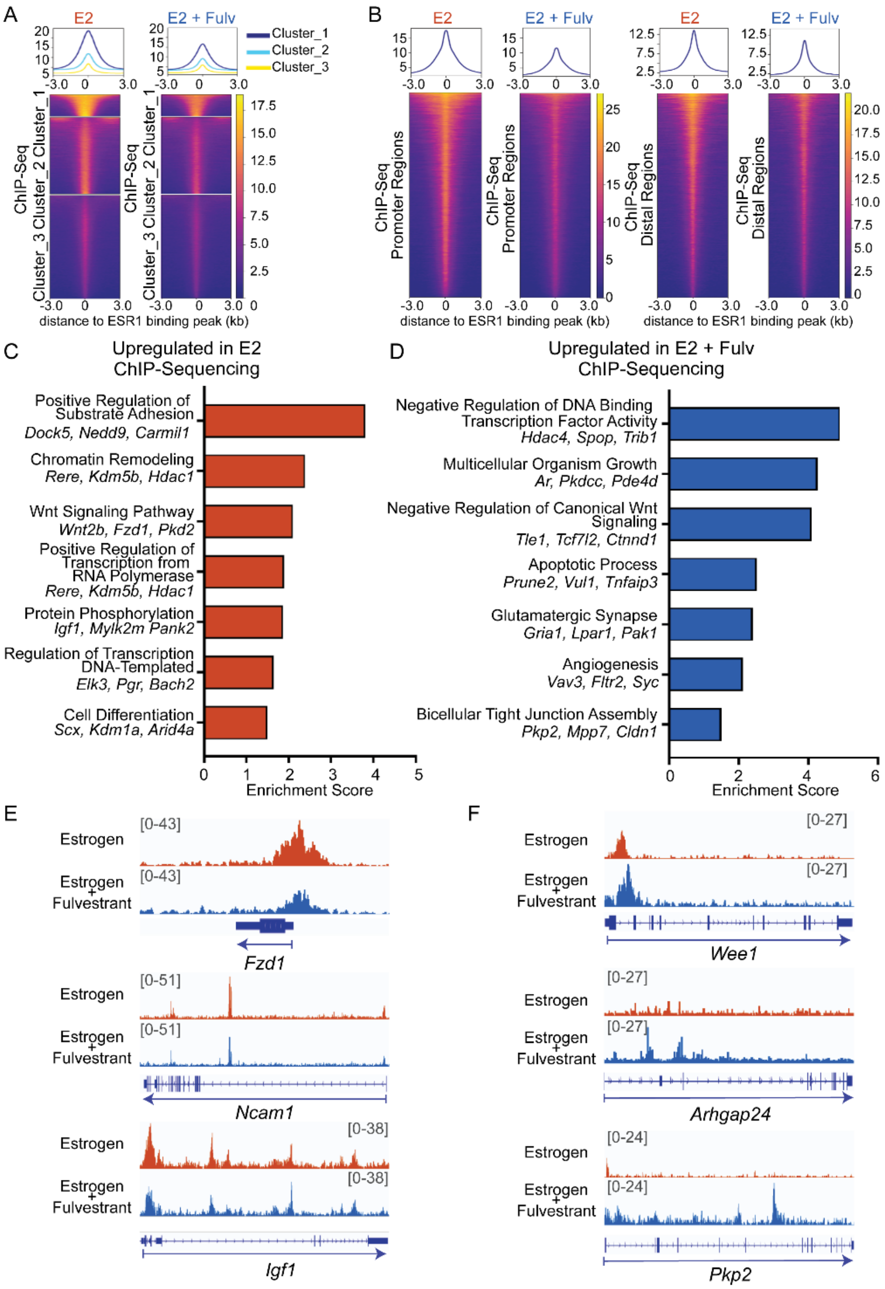

<u>Supplementary Fig. S5: Genomic overview of E2/ESR1 signaling in hernia-associated fibroblasts by ESR1 ChIP-seq analysis.</u>

(A) ESR1 ChIP-seq heatmaps showing signal intensity from all binding events with input signal subtracted and k-means clustering. **(B)** Heatmap of ESR1 ChIP-seq signal intensity from binding events at promoter (left) and distal (right) regions after E2 or E2 + fulvestrant

treatment (n = 3/group). Pathway analysis of top differential peaks enriched in **(C)** E2-treated and **(D)** E2 + Fulvestrant-treated HAFs. Genome browser snapshots of representative genes with stronger peaks in **(E)** E2-treated and in **(F)** E2 + fulvestrant-treated HAFs in ESR1 ChIP-seq analysis. Arrows indicate the direction of the gene transcription in **(E)** and **(F)**.

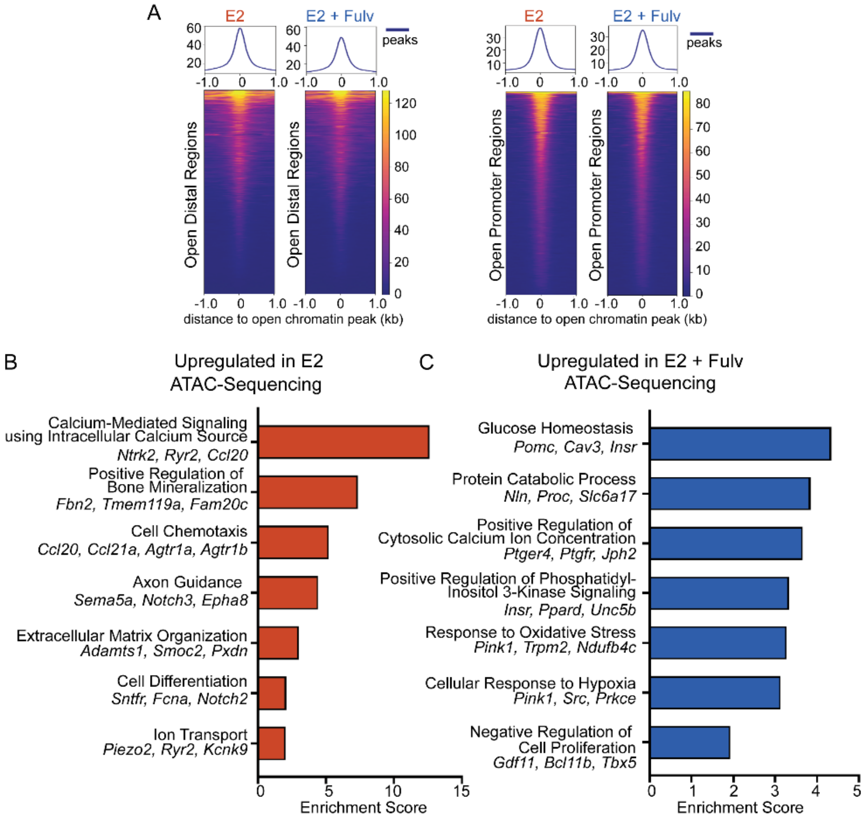

<u>Supplementary Fig. S6: Epigenomic overview of E2/ESR1 signaling in hernia-associated fibroblasts.</u>

(A) Heatmaps of ATAC-seq of HAFs after E2 or E2+fulvestrant treatment, separated by distal and promoter regions. Pathway analysis of top differential peaks enriched in **(B)** E2-treated and **(C)** E2 + fulvestrant-treated HAFs (n = 3/group).

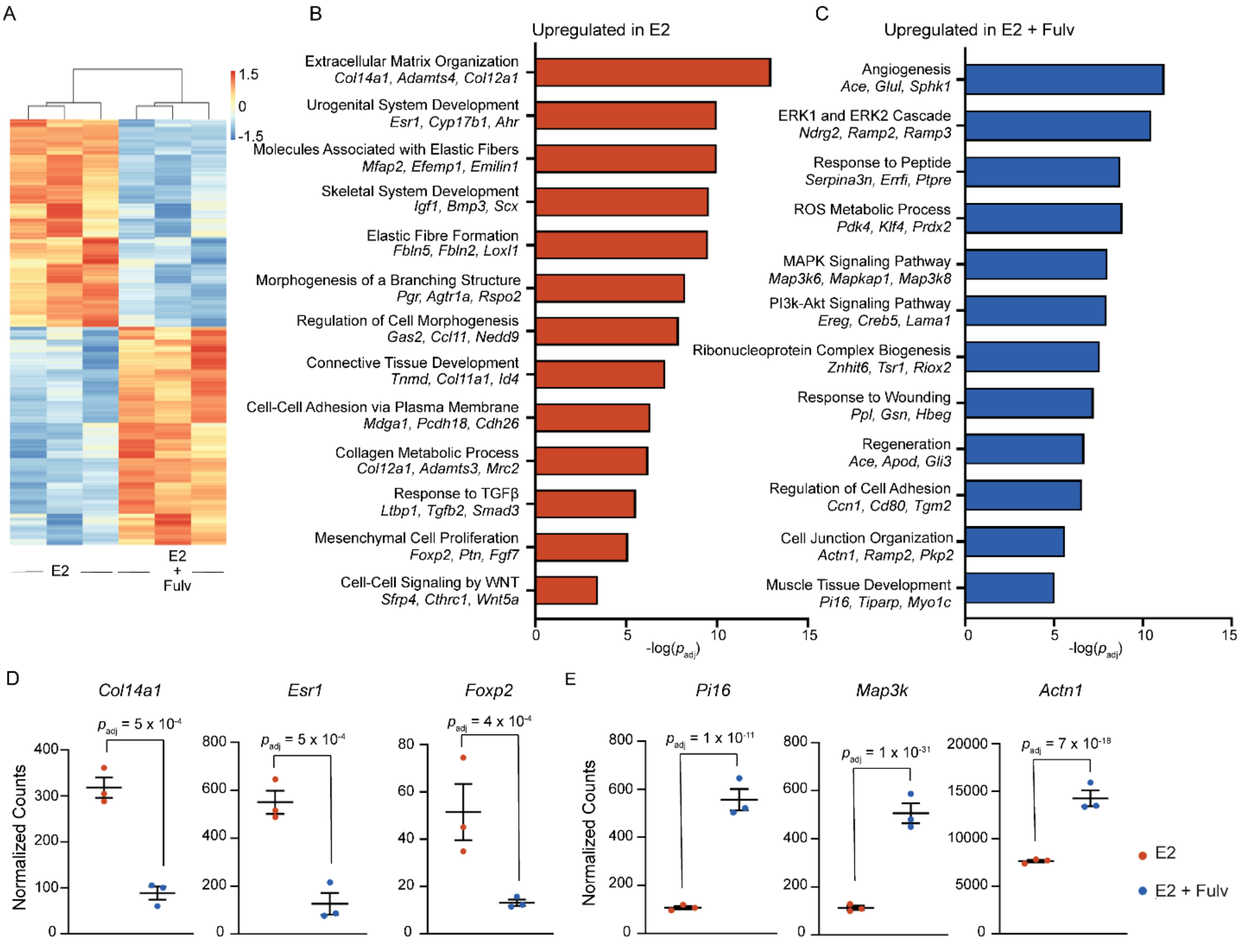

Supplementary Fig. S7: Effect of E2/ESR1 on hernia-associated fibroblast transcriptome.

(A) Heatmap of RNA-seq showing differential gene expression comparing E2 and E2 + fulvestrant-treated LAM HAFs (n = 3/group). Enriched pathways were upregulated in **(B)** E2-treated HAFs or **(C)** in E2 + fulvestrant-treated HAFs, accompanied by top-ranked genes associated with each pathway. Dot plots of RNA-seq counts of the representative genes that were upregulated in **(D)** E2-treated HAFs and **(E)** E2 + fulvestrant-treated HAFs compared with each other (n=3/group).

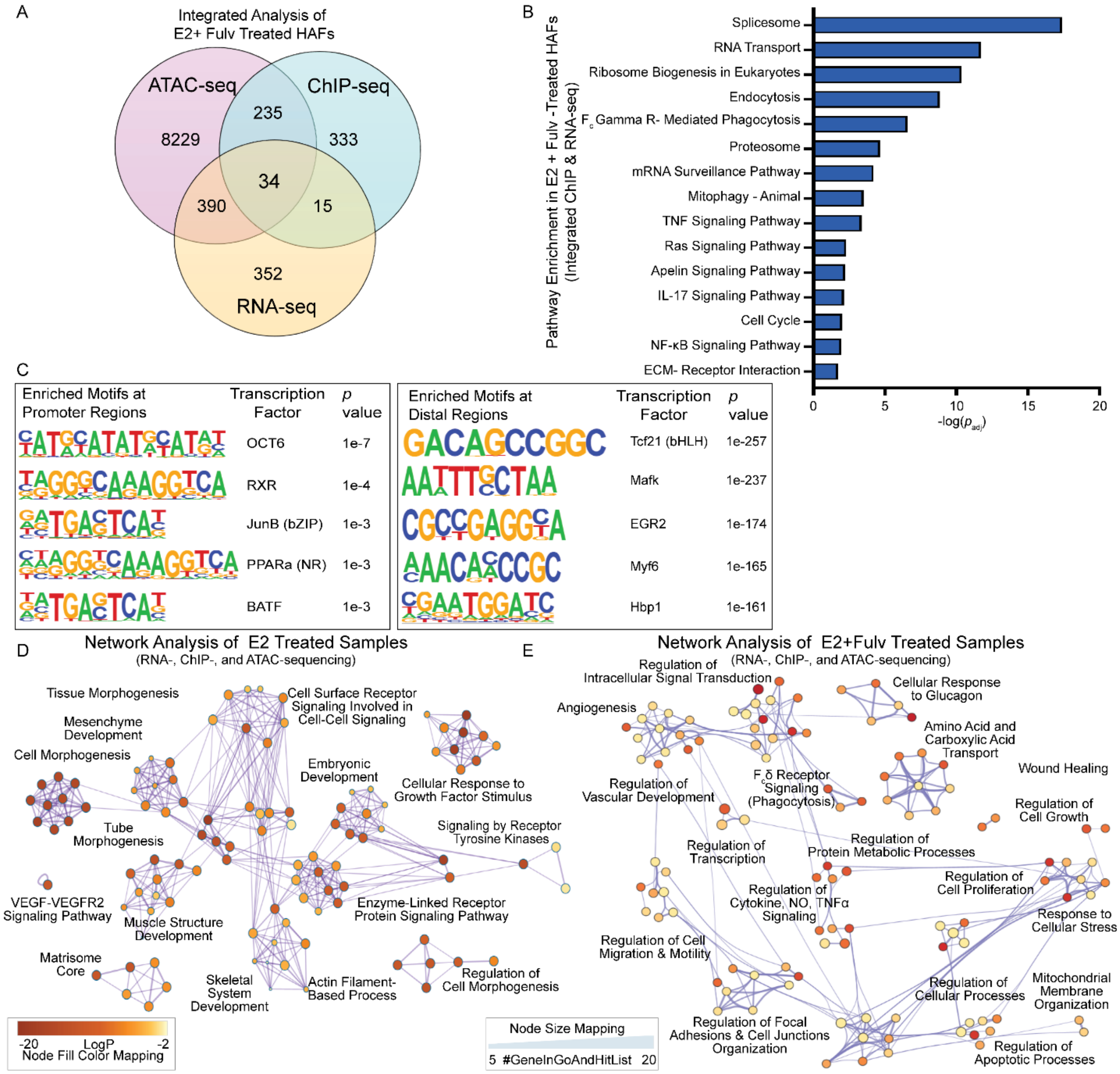

Supplementary Fig. S8: E2/ESR1-related regulatory networks in E2 + fulvestrant treated hernia-associated fibroblasts identified by RNA-seq, ChIP-seq, and ATAC-seq.

(A) Venn diagram showing overlap of genes upregulated with E2 + fulvestrant treatment in the three multi-omics assays, i.e., RNA-seq, ChIP-seq, and ATAC-seq (fold change > 1.2, p<0.05). **(B)** Functional pathway enrichment of upregulated genes in both ChIP-seq and RNA-seq in E2 + fulvestrant-treated HAFs. **(C)** Motifs enriched from both ChIP-seq

and ATAC-seq in E2 + fulvestrant-treated HAFs at the promoter and distal regions. Network of biological pathways upregulated in **(D)** E2-treated and **(E)** E2 + fulvestrant-treated HAFs.

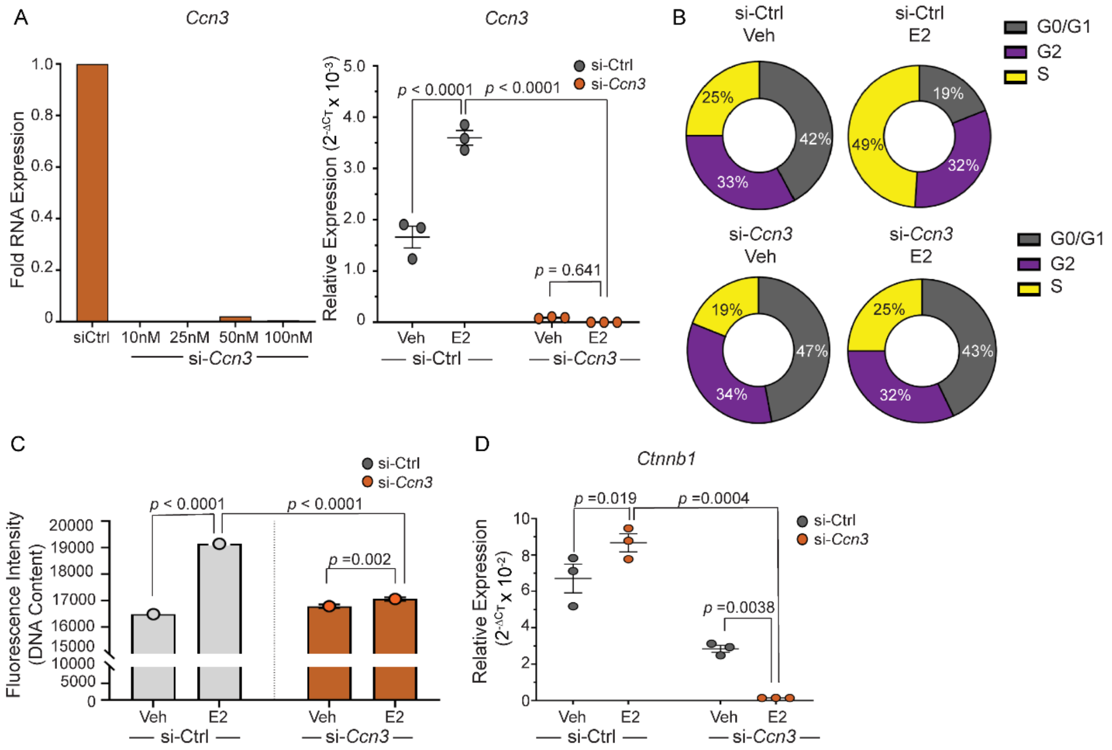

Supplementary Fig. S9: *Ccn3* plays a key role in mediating E2-driven proliferation of HAFs

(A) *Ccn3* RNA expression at various siRNA concentrations (left) and following vehicle or E2 treatment (right, 25 nM si-*Ccn3*) (n = 3, mean ± S.E.M, one-way ANOVA with t-tests for multiple comparisons). **(B)** Distribution of HAFs across cell cycle stages (G0/G1, S, and G2 phases) following *Ccn3* knockdown and E2 treatment (n = 3). **(C)** DNA content in HAFs treated with vehicle or E2, with and without *Ccn3* knockdown (n = 3, mean ± SEM, one-way ANOVA with t-tests for multiple comparisons). **(D)** Expression of beta-catenin (*Ctnnb1*), a key downstream marker of *Ccn3* response, following *Ccn3* knockdown (n = 3, mean ± SEM, one-way ANOVA with t-tests for multiple comparisons).

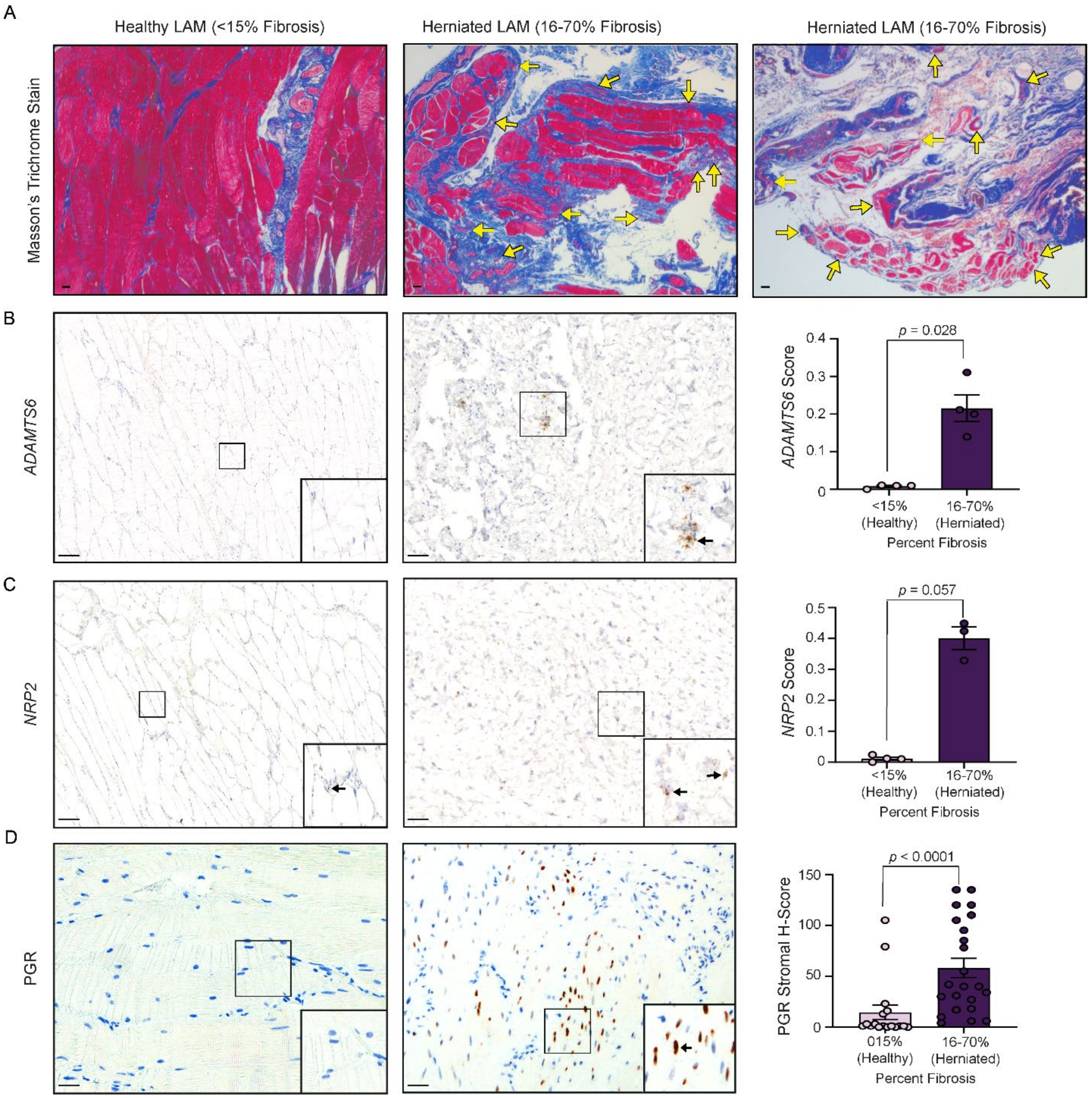

<u>Supplementary Fig. S10: E2/ESR1 modulated genes in men with inguinal hernias</u>

(A) Representative images of Masson's Trichrome stain in tissues from healthy and herniated LAM. Arrows point to atrophied myofibers. RNAscope images of the genes **(B)** *ADAMTS6* and **(C)** *NRP2* identified from multiomic studies that were observed in some patient samples (n = 8 tissues from 4 patients, mean ± S.E.M, t-test, scale bar:

200 μm). **(D)** Immunohistochemical staining of PGR. Black arrows point to positive staining (n = 44 samples from 22 patients, mean ± S.E.M, t-test, scale bar: 200 μm).

METHODS

Immunohistochemistry and Immunocytochemistry

H&E and Masson's Trichrome Staining: LAM from *Arom*^{hum} mice were dissected and fixed in 4% phosphate-buffered paraformaldehyde for 24 hours at 4°C. Tissues were subsequently embedded in paraffin and sectioned at 4-μm thickness. The sections were stained with hematoxylin and eosin (H&E) and Masson's Trichrome (Weigert's Hematoxylin, Biebrich scarlet-acid fuschin solution, and Aniline blue) using a staining kit (American Master Tech, # KTTRBPT). Images were obtained using a Zeiss Axio Scope and EVOS M5000 (Thermo Fisher Scientific) microscope at ×20 magnification (2). The area of fibrosis was quantified using ImageJ v1.53f51 measure function.

Immunocytochemistry: HAFs and NIH/3T3 cells were first washed with PBS and fixed in 4% paraformaldehyde for 10 minutes at room temperature and then permeabilized with 0.5% Triton-X in PBS for 5 minutes. These HAFs were blocked with 5% BSA for 1 hour at room temperature. Primary antibodies (2 μg/mL of PDGFRA [R&D Systems #AF1062], 5 μg/mL of ESR1 [MilliporeSigma #06-935], 1:400 of PBX1 [Invitrogen #PA517223], 1:200 of NCAM1 [Proteintech #142551AP], 1:200 of PIEZO2 [Invitrogen # PA572976], 1:200 of PGR [ABclonal # A0321], or 1:200 of ADAMTS6 [Invitrogen #PA560365]) in wash buffer (1% BSA + 0.1% Tween20) were added to HAFs and incubated overnight at 4°C. Secondary antibodies (Invitrogen #A31573, #A32814, or #A31572) in PBS were added to the samples and incubated for 1 hour in the dark. HAFs were washed 3 times with PBS and incubated in 0.5 μg/mL of DAPI for 5 minutes,

then washed 3 times with wash buffer. Coverslips were mounted onto slides with antifade mountant (Invitrogen #S36937). Images were obtained using an EVOS M5000 microscope (Thermo Fisher Scientific).

ESR1, PDGFRA, PGR, Ki67 Immunohistochemistry: LAM were fixed in 4% PBS-paraformaldehyde solution overnight, embedded in paraffin, and sectioned at 4 µm. After deparaffinization and citrate antigen retrieval (Fisher Scientific, #50843064), sections were incubated in primary antibodies against ESR1 (1:400 Millipore Sigma #06-935 for mouse, 1:100 Biocare SP1 #OAA-301-T60 for human, 1:400 Dako #M3569), PDGFRA (2.5 µg/mL FITC-conjugated Invitrogen 11-1401-82 for mouse, 1:250 Abcam #ab134123 for human), Ki67 (10 µg/mL R&D Systems #AF7649 for mice, 1:50 Dako #M7240 for human), or PGR (1:400, Dako #M3569 for human) overnight at 4°C. After washing, sections were incubated with secondary antibodies (Vector Laboratories for HRP-conjugated). DAB (Dako #GV825) was used for chromogenic staining

Quantification: Scoring was performed with blinding to sample type and treatment by an independent pathologist. For human PDFGRA and ESR1, staining was categorized in to weak, moderate, and strong expression and weighted accordingly to derive the H-score, H score was calculated as [(% weak staining*1) + (% moderate staining*2) + (% strong staining*3)] with scores ranging from 0-300. Ki-67 was quantified as percentage of positive nuclear staining. For mouse studies, percent fibrosis was calculated using Masson's Trichrome staining using ImageJ color deconvolution and measure functions (3).

Collagen Content Measurement

LAM from *Arom*^{hum} and WT mice were harvested and homogenized using a Dounce homogenizer (Active Motif #40401). Collagen content was measured using a hydroxyproline assay kit (Abcam #ab222941) according to the manufacturer’s instructions.

RNAscope™ Assay

Chromogenic *in situ* mRNA detection for transcripts was manually performed on human LAM muscle samples using the RNAscope 2.5 HD Detection kit (ACD Bio, #322300). 5-µm thick formalin-fixed paraffin-embedded tissue sections were pretreated with heat and protease before hybridization. Slides were processed according to the manufacturer’s instructions with some modifications: hydrogen peroxide treatment for 30 minutes, AMP 5 hybridization for 45 minutes, and AMP 6 hybridization for 22.5 minutes. Tissue sections were hybridized with RNAscope target probes. Probes to the *DapB* bacterial gene (probe DapB cat# 310043) and the endogenous human *UBC* mRNA (probe #310041) were used as technical negative and positive controls, respectively, for each run. Positive mRNA expression was demonstrated by brown, punctate staining present within the cytoplasm and/or nucleus. The probes were purchased from ACD (Hs-NCAM1 [#421461, Accession No: NM_001242608.1], Hs-LTBP1 [#523281, Accession No: NM_000627.3], Hs-ADAMTS6 [#814831, Accession No: NM_197941.4], Hs-NRP2 [#422371, Accession No: NM_201264.1], Hs-PBX1 [#490041, Accession No: NM_002585.3], and Hs-PIEZO2 [#449951, Accession No: NM_022068.3]). Probe signal was quantified using ImageJ “Trainable Weka Segmentation” plugin (4).

Table S1. Significantly upregulated genes in HAFs after E2 treatment across the RNA-seq, ChIP-seq, and ATAC-seq datasets

Gene	Name
Mrc2	Mannose Receptor C Type 2
Tmem86a	Transmembrane Protein 86A
Npas3	Neuronal PAS domain-containing protein 3
Gcnt1	Beta-1,3-galactosyl-O-glycosyl-glycoprotein beta-1,6-N-acetylglucosaminyltransferase
Rbp1	Retinol-binding protein 1
Adcy3	Adenylate cyclase type 3
Lipe	Hormone-sensitive lipase
Adamts3	a disintegrin-like and metallopeptidase with thrombospondin type 1 motif, 3
Svil	Supervillin
Adgra2	Adhesion G protein-coupled receptor A2
Obsl1	Obscurin-like protein 1
Adamts6	A disintegrin-like and metallopeptidase with thrombospondin type 1 motif, 6
Prkd3	Pyruvate Dehydrogenase Kinase 3
Ccn3	Cellular communication network factor 3
Mgat3	Beta-1,4-mannosyl-glycoprotein 4-beta-N-acetylglucosaminyltransferase
Ogdhl	Oxoglutarate dehydrogenase (succinyl-transferring)
Cdon	Cell adhesion molecule-related/down-regulated by oncogenes
Fxyd1	Phospholemman
Prickle2	Prickle Planar Cell Polarity Protein 2
Kcnma1	Calcium-activated potassium channel subunit alpha-1
Id2	DNA-binding protein inhibitor ID-2
Ltbp1	Latent-transforming growth factor beta-binding protein 1
Pdcd4	Programmed cell death protein 4
Pcdh7	Protocadherin-7
Nrp2	Neuropilin-2
Kif26b	Kinesin-like protein KIF26B
Chst2	Carbohydrate sulfotransferase 2
Aff3	AF4/FMR2 family member 3
Kalrn	Kalirin
Pgr	Progesterone receptor
Zfp521	Zinc finger protein 521
Igfbp4	Insulin-like growth factor-binding protein 4
Tanc2	Tetratricopeptide repeat, ankyrin repeat and coiled-coil domain-containing protein 2
Fbln5	Fibulin-5
Hlf	Hepatic leukemia factor
Fbln7	Fibulin-7
Mex3a	Mex3 RNA-binding family member A
Pbx1	Pre-B-cell leukemia transcription factor 1
Piezo2	Piezo-type mechanosensitive ion channel component 2
Pde8b	High affinity cAMP-specific and IBMX-insensitive 3',5'-cyclic phosphodiesterase 8B
Maml3	Mastermind Like Transcriptional Coactivator 3
Cntln	Centlein
Bach2	BTB Domain And CNC Homolog 2
Ncam1	Neural cell adhesion molecule 1
Rnf150	RING finger protein 150
Zfp618	Zinc finger protein 618
Plcl1	Inactive phospholipase C-like protein 1
Cdc42ep5	Cdc42 effector protein 5
Apcdd1	Protein APCDD1
Ssbp2	Single-stranded DNA-binding protein 2
Pknox2	Homeobox protein PKNOX2
Ntn4	Netrin-4

Spats2l	Spermatogenesis Associated Serine Rich 2 Like
Gpr85	Probable G-protein coupled receptor 85
Tnik	Traf2 and NCK-interacting protein kinase
Cxxc5	CXXC-type zinc finger protein 5
Twsg1	Twisted gastrulation protein homolog 1
Wnt5a	Protein Wnt-5a

Table S2. Significantly upregulated genes in HAFs after E2 plus fulvestrant treatment across the RNA-seq, ChIP-seq, and ATAC-seq datasets

Gene	Name
Gbe1	1,4-alpha-glucan-branching enzyme
Trim24	Transcription intermediary factor 1-alpha
Ppm1h	Protein phosphatase 1H
Avpr1a	Vasopressin V1a receptor
Selenop	Selenoprotein P
Stim1	Stromal interaction molecule 1
Col4a2	Collagen alpha-2(IV) chain
Gsn	Gelsolin
Trib1	Tribbles homolog 1, TRB-1
Plcxd2	PI-PLC X domain-containing protein 2
Plce1	1-phosphatidylinositol 4,5-bisphosphate phosphodiesterase epsilon-1
Cdkn1c	Cyclin-dependent kinase inhibitor 1C
Ampd3	AMP deaminase 3
Akap13	A-kinase anchor protein 13
Mb21d2	Nucleotidyltransferase MB21D2
Higd1a	HIG1 domain family member 1A, mitochondrial
Ccnl1	Cyclin-L1
Wee1	Wee1-like protein kinase
Dcbld2	Discoidin, CUB and LCCL domain-containing protein 2
Cdc7	Cell division cycle 7-related protein kinase
Dlgap1	Disks large-associated protein
Klf15	Krueppel-like factor 15
Sorbs1	Sorbin and SH3 domain-containing protein 1
Nav3	Neuron navigator 3
Arhgap24	Rho GTPase-activating protein 24
Pdk4	Pyruvate dehydrogenase (acetyl-transferring)] kinase isozyme 4, mitochondrial
Fkbp5	Peptidyl-prolyl cis-trans isomerase FKBP5
Nfil3	Nuclear factor interleukin-3-regulated protein
Dusp14	Dual specificity protein phosphatase 14
Pkp2	Plakophilin-2
Aff1	AF4/FMR2 family member 1,
Slc7a1	High affinity cationic amino acid transporter 1
Dpep1	Dipeptidase 1
Gda	Guanine deaminase

REFERENCES

1. Potluri T, Taylor MJ, Stulberg JJ, Lieber RL, Zhao H, and Bulun SE. An estrogen-sensitive fibroblast population drives abdominal muscle fibrosis in an inguinal hernia mouse model. *JCI insight.* 2022;7(9).
2. Zhao H, Zhou L, Li L, Coon VJ, Chatterton RT, Brooks DC, et al. Shift from androgen to estrogen action causes abdominal muscle fibrosis, atrophy, and

inguinal hernia in a transgenic male mouse model. *Proc Natl Acad Sci U S A.* 2018;115(44):E10427-E36.
3. Chen Y, Yu Q, and Xu C-B. A convenient method for quantifying collagen fibers in atherosclerotic lesions by ImageJ software. *Int J Clin Exp Med.* 2017;10(10):14904-10.
4. Arganda-Carreras I, Kaynig V, Rueden C, Eliceiri KW, Schindelin J, Cardona A, et al. Trainable Weka Segmentation: a machine learning tool for microscopy pixel classification. *Bioinformatics.* 2017;33(15):2424-6.
